# An Update on *Trichoderma* Mitogenomes: Complete *De Novo* Mitochondrial Genome of the Fungal Biocontrol Agent *Trichoderma harzianum* (Hypocreales, Sordariomycetes), an Ex-Neotype Strain CBS 226.95, and Tracing the Evolutionary Divergences of Mitogenomes in *Trichoderma*

**DOI:** 10.3390/microorganisms9081564

**Published:** 2021-07-23

**Authors:** Yunyoung Kwak

**Affiliations:** 1 Écologie, Systématique et Évolution, CNRS, Université Paris Sud (Paris XI), Université Paris Saclay, AgroParisTech, 91400 Orsay, France; yun@knu.ac.kr; 2 School of Applied Biosciences, Kyungpook National University, Daegu 41566, Korea; 3 Institute for Quality and Safety Assessment of Agricultural Products, Kyungpook National University, Daegu 41566, Korea

**Keywords:** divergence, divergence time, evolution, fungal mitochondrial genome, fungal mitogenome, selective pressure, *Trichoderma*

## Abstract

Members of the genus *Trichoderma* (Hypocreales), widely used as biofungicides, biofertilizers, and as model fungi for the industrial production of CAZymes, have actively been studied for the applications of their biological functions. Recently, the study of the nuclear genomes of *Trichoderma* has expanded in the directions of adaptation and evolution to gain a better understanding of their ecological traits. However, *Trichoderma*’s mitochondria have received much less attention despite mitochondria being the most necessary element for sustaining cell life. In this study, a mitogenome of the fungus *Trichoderma harzianum* CBS 226.95 was assembled *de novo.* A 27,632 bp circular DNA molecule was revealed with specific features, such as the intronless of all core PCGs, one homing endonuclease, and a putative overlapping tRNA, on a closer phylogenetic relationship with *T. reesei* among hypocrealean fungi. Interestingly, the mitogenome of *T. harzianum* CBS 226.95 was predicted to have evolved earlier than those of other *Trichoderma* species and also assumed with a selection pressure in the *cox3*. Considering the bioavailability, both for the ex-neotype strain of the *T. harzianum* species complex and the most globally representative commercial fungal biocontrol agent, our results on the *T. harzianum* CBS 226.95 mitogenome provide crucial information which will be helpful criteria in future studies on *Trichoderma*.

## 1. Introduction

Mitochondria are essential double-membrane bound subcellular compartments responsible for producing cellular energy, adenosine triphosphate (ATP), in eukaryotes [1]. Typically, their genetic materials exist in the form of a single chromosome (i.e., mitochondrial genome or mitogenome) [2] and consist of (1) conserved core protein-coding genes (PCGs) for the functional proteins involved in oxidative phosphorylation (OXPHOS), (2) genes encoding small- and large subunits of ribosomal RNA (rRNAs), and (3) transfer RNAs (tRNAs) [3]. However, mitogenomic features (e.g., lengths, gene orders, intergenic-/repetitive regions, mobile elements, and tRNA distribution) have been characterized differently in various eukaryotic lineages [4].

Mitogenomes are ideal tools for the evolutionary analysis of eukaryotes due to their (1) small size, (2) high copy numbers, (3) limited recombination, and (4) high mutation rates leading to independent evolutions of nuclear genomes [2,5]. When considering the ecological features of fungi, such as a broad diversity of lineages and lifestyles in nature, fungal mitogenomes can provide crucial information on evolutionary adaptations and divergences of the mitochondria in different environments [1,6]. According to the recent rapid developments of high-throughput sequencing technologies, fungal organelle genome databases have increasingly accumulated, however, these are still less abundant than those of animals and plants [4,7]. Thus far, most studies of fungal mitogenomes have focused on the (1) sequencing and characterization of individuals and (2) the elucidation of their phylogenetic positions within its relatives to support the potentials for one of the significant molecular markers identifying fungal species. Until recently, few studies have reported on comparative analyses of mitogenomes, ranging from a taxa level for specific fungal groups, under evolutionary viewpoints [8,9,10,11].

*Trichoderma* spp. (Hypocreales, Sordariomycetes, Ascomycota) are widely known as representative fungal biocontrol agents of plant pathogens due to their diverse nutritional modes (e.g., for being as saprotrophic, endophytic, and mycoparasitic) [12,13,14]. Since the genome sequencing of *T. reesei* QM6a (GenBank accession no. AAIL00000000) was first presented [15], *Trichoderma* sequencing studies have actively increased to gain a better understanding of their biological and ecological roles for the more improved applications (e.g., biofungicide, biofertilizer, and a producer of plant biomass hydrolyzing enzymes (CAZymes)); however, they were mainly focused on nuclear genomes [16,17,18,19,20]. To date (as of January 2021), only five *Trichoderma* mitogenomes (*T. asperellum* B05 (GenBank accession no. NC_037075), *T. atroviride* ATCC 26799 (GenBank accession no. MN125601), *T. gamsii* KUC1747 (GenBank accession no. KU687109), *T. hamatum* (GenBank accession no. MF287973), and *T. reesei* QM9414 (GenBank accession no. AF447590)) are available as a verified dataset in GenBank. In our previous report of the *T. atroviride* ATCC 26799 mitogenome, all of these five complete sequences were compared and reviewed for the first time [11].

When considering the species complex of *T. harzianum*, initially it was suggested to be an aggregate species including several cryptic species [21], genomic studies of *T.* *harzianum* can provide more knowledge of their adaptive evolution and functional mechanisms in particular niches with both of systematic- and ecological perspectives [19,20]. In terms of the *T. harzianum* CBS 226.95, an ex-neotype strain of the *T. harzianum* species complex and the most globally representative commercial fungal biocontrol agent, the nuclear genome of this fungus has already been sequenced (GenBank accession no. GCA_003025095) [19], but the mitogenome has not. Here, we present a complete *de novo* mitogenome of the fungus *T. harzianum* CBS 226.95 with genomic characterization, comparative analyses, and the tracing of time-scaled evolutionary divergences.

## 2. Materials and Methods

### 2.1. DNA Extraction and Genome Sequencing

The ex-neotype strain *T. harzianum* CBS 226.95 [22] was obtained from the Westerdijk Fungal Biodiversity Institute (formerly the Centraalbureau voor Schimmelcultures (CBS) Fungal Biodiversity Centre, The Netherlands). The fungus was cultured on Difco^TM^ potato dextrose medium (Difco Laboratories Inc., Detroit, MI, USA) at 25 °C, and the obtained mycelia were used as a source of genomic DNA. Total genomic DNA was extracted using a Plant/Fungi DNA Isolation Kit (Sigma-Aldrich Co. Ltd., St. Louis, MO, USA) according to the manufacturer’s instructions and followed by further purification using Phenol-Chloroform (Sigma-Aldrich Co. Ltd., USA) [23]. A quality/quantity of the extracted DNA was measured using the Qubit assay in a Qubit^TM^ 3.0 Fluorometer (Thermo Fisher Scientific Inc., Waltham, MA, USA).

The genomic DNA was used to construct a 20 kb insert SMRTbell^®^ DNA library in a BluePippin^TM^ size-selection system (Pacific Biosciences, Menlo Park, CA, USA). Then, it was sequenced on a single molecule real-time (SMRT) sequencing platform by a PacBio RS-II DNA sequencer with P6 polymerase-C4 sequencing chemistry (Pacific Biosciences, USA) [24] at the Génome Québec Innovation Centre (Montréal, QC, Canada).

### 2.2. De Novo Assembly and Annotation

Only raw reads with a read-quality higher than 0.85 were used for *de novo* mitogenome assembly using the hierarchical genome-assembly process (HGAP) [25] in the SMRT^TM^ pipeline (Pacific Biosciences, USA). The *de novo* mitogenome assembly’s accuracy was verified using the P-mapping module [26], and the circularity was confirmed using Gepard [27].

The mitogenome was primarily annotated on webservers for Mfannot (Mfannot, http://megasun.bch.umontreal.ca/cgi-bin/mfannot/mfannotInterface.pl (accessed on 12 January 2021) [28] and MITOS (Mitos, http://mitos.bioinf.uni-leipzig.de/index.py (accessed on 12 January 2021)) [29], under the Genetic Code 4 (the Mold, Protozoan, and Coelenterate Mitochondrial Code). Then, these annotations were modified by BLAST searching (default parameters) on the NCBI non-redundant (nr) database [30] and NCBI Open Reading Frame (ORF) Finder [31]. All annotations relating to gene boundaries were also manually curated to avoid artificial frameshifts in the ORFs. The tRNAs were annotated using the webserver for RNAweasel (RNAweasel, http://megasun.bch.umontreal.ca/cgi-bin/RNAweasel/RNAweaselInterface.pl (accessed on 12 January 2021)) [32] and tRNAScan-SE (v.2.0) [33], under the Genetic Code 4. A circular map of the mitogenome was illustrated using Circos [34]. Finally, the complete *de novo* mitogenome sequence of *T. harzianum* CBS 226.95 was deposited in GenBank under the accession number MN564945.

### 2.3. Genomic Analysis

The base composition and codon usage were analyzed using MEGA (v.7.0) [35] and the EMBOSS package [36]. The secondary structure of tRNAs was predicted by tRNAScan-SE (v.2.0) [33], and the asymmetric bias of nucleotide composition was calculated using the following formulas [37]: AT-skew = (A − T)/(A + T) and GC-skew = (G − C)/(G + C). The interspersed-/tandem repeats were analyzed using BLAST [38] via the self-comparison of mitogenome itself (parameter: E-values < 10^−10^) and Tandem Repeats Finder (v.4.0; default parameters) [39], respectively. All intron loci and intron types were investigated on the webserver for RNAweasel (RNAweasel, http://megasun.bch.umontreal.ca/cgi-bin/RNAweasel/RNAweaselInterface.pl (accessed on 12 January 2021)) [32] under the Genetic Code 4, and intronic ORFs were identified by BLAST searching on the NCBI-nr database [30] and NCBI-ORF Finder [31]. Finally, the evolutionary pairwise genetic distance between the nucleotide sequences was measured using the proportional (p) distance method in MEGA (v.7.0) [35], and the obtained values were plotted using ggplot2 [40] in R (v.3.4) [41].

### 2.4. Phylogenetic Analyses

A total of 47 Sordariomycetes mitogenomes were downloaded from the GenBank database on NCBI (GenBank, https://www.ncbi.nlm.nih.gov/genbank/ (accessed on 12 January 2021)) [42]. Full nucleotide sequences of the 13 core PCGs (*atp6*, *atp8*, *cob*, *cox1*, *cox2*, *cox3*, *nad1*, *nad2*, *nad3*, *nad4*, *nad4L*, *nad5*, and *nad6*; in this study, the *atp9* gene was excluded because it has not been reported in the GenBank dataset of the *T. gamsii* KUC1747 mitogenome (GenBank accession no. KU687109)) were individually aligned using MAFFT (v.7.4; parameter: mafft -auto) [43], and these multiple alignments were concatenated in SequenceMatrix (v.1.8) [44]. The best-fit evolutionary model was determined using jModelTest (v.2.1) under the Information Criterion of Akaike (AIC) and Bayesian (BIC) [45], and the GTR + Gamma + I substitution model was selected as the best-fit model from all criteria.

Maximum likelihood (ML) [46] analysis was conducted in RAxML (v.8.2) with 1000 bootstrap replicates [47], and the bootstrap (BS) values were displayed on the nodes of the constructed phylogenetic tree. Bayesian inference (BI) [48,49] analysis was conducted in MrBayes (v.3.2) [50] under the Markov chains Monte Carlo (MCMC) algorithm (three heated and one cold chain, with a heating coefficient 0.1) for 2 × 10^6^ generations (sampling for every 400 gen.). After a burn-in for the first 25% of the sampled trees, the remaining trees were used to construct the consensus BI tree with values of Bayesian posterior probabilities (BPPs). The stationarity of BI analysis was determined by calculating the average standard deviation for split frequencies (<0.01).

### 2.5. Time-Scaled Bayesian Phylogeny

Using the target mitogenomes of Hypocreales species, only exon sequences of the 13 core PCGs (*atp6*, *atp8*, *cob*, *cox1*, *cox2*, *cox3*, *nad1*, *nad2*, *nad3*, *nad4*, *nad4L*, *nad5*, and *nad6*; the *atp9* gene was excluded because it has not been reported in the GenBank dataset of the *T. gamsii* KUC1747 mitogenome (GenBank accession no. KU687109)) were individually aligned using MAFFT (v.7.4; parameter: mafft -auto) [43] and concatenated in SequenceMatrix (v.1.8) [44].

Under a best-fit of the GTR + Gamma substitution model determined by jModelTest (v.2.1) [45], MCMC analysis was counted for 4 × 10^7^ generations (sampling for every 1000 gen.) with a lognormal relaxed clock in BEAST2 (v.2.5) [51]. Calibration nodes were defined by five ancestral nodes (i.e., set clade ages for covering a central 95% probability range at the common ancestral node for (1) all species (272–288 Mya); (2) the order Hypocreales (185–201 Mya); (3) the family Nectriaceae (117–133 Mya); (4) the family Ophiocordycipitacea (109–125 Mya); and (5) the family Hypocreaceae (112–128 Mya)—based on the Bayesian chronogram previously reported using 638 core orthologous proteins by Hypocreales genomes [20], and species within the clades for each calibration node were constrained to be a monophyletic group. All parameters of MCMC output were verified to meet the effective sample size (ESS; as above 200) in Tracer (v.1.7) [52]. After a burn-in for the first 25% of the sampled trees, only remaining trees were used to construct the consensus tree in TreeAnnotator (v.2.5; in BEAST2 package), and all estimated divergence times (million years ago, Mya) were visualized on the time-scaled BI tree for node ages in FigTree (FigTree, http://tree.bio.ed.ac.uk/software/figtree/ (accessed on 12 January 2021)) (v.1.4).

### 2.6. Selective Pressures Analysis

The exon sequences of the 13 core PCGs (*atp6*, *atp8*, *cob*, *cox1*, *cox2*, *cox3*, *nad1*, *nad2*, *nad3*, *nad4*, *nad4L*, *nad5*, and *nad6*; the *atp9* gene was excluded because it has not been reported in the GenBank dataset of the *T. gamsii* KUC1747 mitogenome (GenBank accession no. KU687109)) were individually aligned using ClustalW (parameter: -codon option) in MEGA (v.7.0) [35], and these in-frame codon alignments were used to explore evolutionary pressures.

The non-synonymous substitution rate (Ka), synonymous substitution rate (Ks), and Ka/Ks ratio were calculated on the pairwise alignments of each target gene between species using DnaSP (v.6.12) [53], and obtained values were plotted using ggplot2 [40] in R (v.3.4) [41]. The codon sites evolving under the episodic diversifying positive selection were detected using the mixed effects model of evolution (MEME) method [54] in HyPhy (v.2.1) [55], and sites under the *p*-value threshold of 0.1 (*p* < 0.1) were considered.

## 3. Results

### 3.1. Genomic Features of the T. harzianum CBS 226.95 Mitogenome

After filtering low-quality reads, a total of 1,806,923,812 read bases were used as seeds for the assembling process, and a complete mitogenome of *T. harzianum* CBS 226.95 was assembled *de novo* with a length of 27,632 bp (coverage depth, 573.59×; GenBank accession no. MN564945).

**Figure 1:**
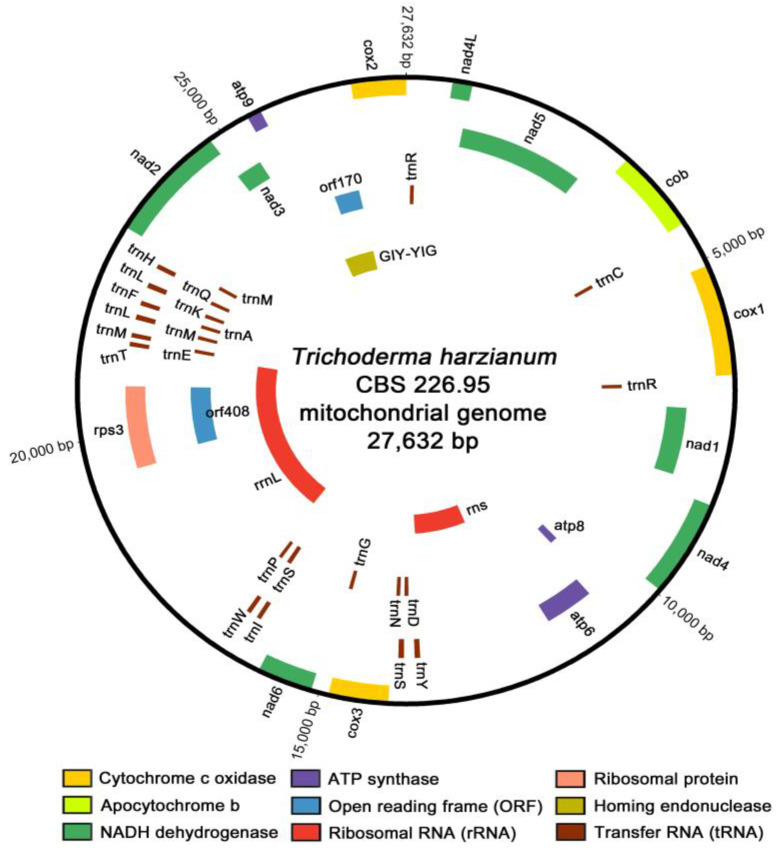
Circular map of the complete mitochondrial genome of *T. harzianum* CBS 226.95. Gene components were indicated with different color blocks, and the circular map was plotted using Circos.

A closed-circular DNA molecule of the *T. harzianum* CBS 226.95 mitogenome was composed of A (36.1%), T (36.4%), G (15.1%), and C (12.4%), with the AT content (72.45%) approximately 2.63 times higher than the GC content. The asymmetric biases of these nucleotide compositions were inferred from the skewness, each for the negative value of AT-skew and the positive value of GC-skew, it showed more frequency of thymine (T) and guanine (G) than adenine (A) and cytosine (C) in the forward strand. The coding and non-coding regions of the entire length of the mitogenome accounted for approximately 79.25% and 20.75%, respectively (Table S1). All genes constituting the coding regions were predicted to be transcribed in a clockwise direction on the same forward strand (Figure 1).

All 14 conserved core PCGs were composed of three ATP synthases (*atp6*, *atp8* and *atp9*), one apocytochrome *b* (*cob*), three cytochrome *c* oxidases (*cox1*, *cox2* and *cox3*), and seven NADH dehydrogenases (*nad1*, *nad2*, *nad3*, *nad4*, *nad4L*, *nad5* and *nad6*), without any introns capable of splitting each of their coding sequences (CDS) to the exon–intron structure. Two ORFs that were predicted without assigned functions, *orf408* and *orf170*, were also found as a form of non-freestanding ORF. The *orf408* gene was entirely located within a coding region of the *rps3* gene for ribosomal protein S3 and *rrnL* gene for a large subunit ribosomal RNA, and the *orf170* gene was identified completely within a conserved domain for homing endonuclease (HE). All of the core PCGs and ORFs were initiated with the standard ATG start codon and terminated with the TAA stop codon, except for three genes: the *nad4* gene with the GTG start codon, and *atp9* and *orf170* with the TAG stop codon. An overlap of one nucleotide base was only found between the TAA stop codon of the *nad4L* gene and the ATG start codon of the *nad5* gene (−1 bp, corresponding to the codon of A at nucleotide position 897) (Table S1).

All 24 tRNA genes (*trn* genes), accounting for approximately 6.49% of the entire mitogenome, were shown with average lengths ranging from 71 bp to 87 bp. These *trn* genes were found as single copies, but several were found in multiple copies with different anticodons (two copies for *trnR*^Arg^ (*trnR*^Arg^[ACG] and *trnR*^Arg^[TCT]), two copies for *trnS*^Ser^ (*trnS*^Ser^[GCT] and *trnS*^Ser^[TGA]), and two copies for *trnL*^Leu^ (*trnL*^Leu^[TAA] and *trnL*^Leu^[TAG])). A *trnM*^Met^ gene coding for methionine was also found in three copies with the same anticodon ((*trnM*^Met^[CAT]) (Tables S1 and S2)). Most of the tRNA genes were predicted to have a typical cloverleaf secondary structure. However, some *trn* genes (positions: *trnY*^Tyr^ at 13,598, *trnS*^Ser^ at 13,854 and 16,306, and *trnL*^Leu^ at 21,911 and 22,462) were found with an extra variable arm (V-arm) (Figure S1), which was consistent with previous reports about the specificity of extra V-arm to tRNA^Leu^, tRNA^Ser^, and bacterial-/eukaryotic organellar tRNA^Tyr^ [56,57].

Two rRNA genes, the *rns* gene for small subunit ribosomal RNA and the *rrnL* gene for large subunit ribosomal RNA, accounted for approximately 22.50% of the entire length of mitogenome. The *rns* gene located between the *atp6* gene and the *cox3* gene was found as a freestanding ORF (length 1503 bp). Otherwise, the *rrnL* gene was predicted in a position ranging over two of PCGs, *orf408* and *rps3* (length 4713 bp). The *rps3* gene for ribosomal protein S3 was found to be an internal ORF of the group I intron that was positioned within the *rrnL* gene [58], and was 1368 bp in size (141 bp larger than *orf408*) with the ATA start codon (Figure 1 and Table S1).

Leucine was the most abundant amino acid (13.57%), followed by isoleucine (11.22%), serine (8.30%), and phenylalanine (7.20%). In contrast, the least frequent amino acid was cysteine (0.46%) (Table S3). Lastly, three specific sites were found for interspersed repeats, and tandem repeats were also detected with consensus lengths ranging from 9 to 26 bp on the multi-copies (Table S4).

### 3.2. Phylogenetic Analyses of the T. harzianum CBS 226.95 Mitogenome

Excluding an unverified dataset, 47 complete mitogenomes (i.e., 46 from Hypocreales species (belonging to the class Sordariomycetes) and one of *N. crassa* OR74A (belonging to the order Sordariales that composed of the class Sordariomycetes with the order Hypocreales)) were retrieved from the GenBank and used in the analyses (Table S5).

**Figure 2:**
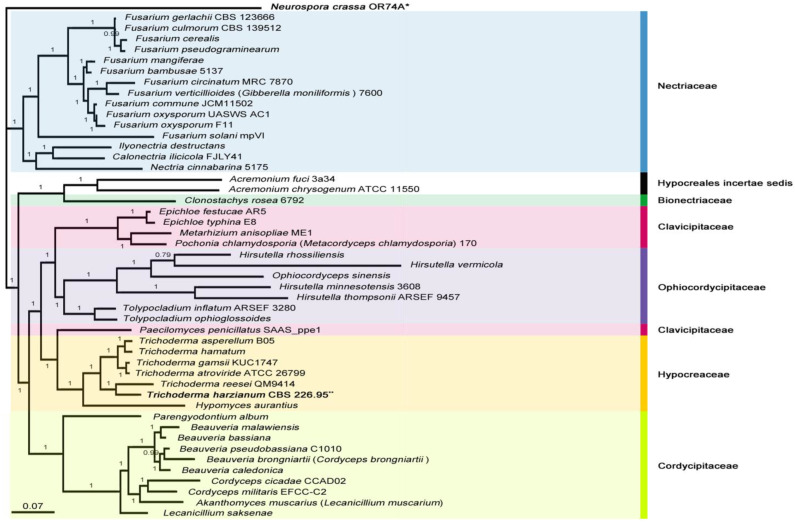
Bayesian phylogeny for a position of the *T. harzianum* CBS 226.95 mitogenome in Hypocreales. The tree was generated using concatenated sequences of 13 core genes (*atp6*, *atp8*, *cob*, *cox1*, *cox2*, *cox3*, *nad1*, *nad2*, *nad3*, *nad4*, *nad4L*, *nad5* and *nad6*), and the mitochondrial genome of *Neurospora crassa* OR74A (Sordariales) was used as an outgroup. All Sordariomycetes species that used for the phylogenetic tree are described in Table S5, and Bayesian posterior probabilities (BPPs) values were marked on the nodes. Asterisks for the *Neurospora crassa* OR74A mitogenome (*) and *T. harzianum* CBS 226.95 mitogenome (**).

In both of the molecular phylogenies (ML- and BI tree), all Hypocreales species were well observed with high confidence, under the significant clades supported by seven major families of the order Hypocreales; interestingly, *Paecilomyces penicillatus* SAAS_ppe1, belonging to the family Clavicipitaceae, was found with a closer relationship with the family Hypocreaceae than those of native family (BS = 100, BPP = 1.00). It may be resolved by improving the Hypocreales phylogeny resolution on accumulations of informative datasets of the mitochondrial genomes from closely related family species (Figure 2 and Figure S2).

The genus *Trichoderma*, including *T. harzianum* CBS 226.95, was placed in a sister clade with the genus *Hypomyces*. Among hypocrealean fungi, a sister relationship between *T. harzianum* ATCC 26799 and *T. gamsii* KUC1747 was observed identically in both tree topologies (BS = 100, BPP = 1.00) (Figure 2 and Figure S2).

### 3.3. Comparative Analysis of the Trichoderma Mitogenomes

The complete *de novo* mitogenome of *T.* *harzianum* CBS 226.95 was compared with five complete *Trichoderma* mitogenomes that publicly available from the genus *Trichoderma,* excluding an unverified dataset (as of January 2021) (Table 1).

The entire lengths of mitogenome ranged from 27,632 bp (*T. harzianum* CBS 226.95) to 42,130 bp (*T. reesei* QM9414) with an average AT content (72.2%). The intergenic regions ranged from 17.58% (*T. reesei* QM9414) to 31.73% (*T. asperellum* B05), and the mitogenome of *T. harzianum* CBS 226.95 was shown to fall in the middle of this range. The mitogenomes of *T. gamsii* KUC1747, *T. asperellum* B05, and *T. hamatum* were presented with the negative value of AT-skew as those of *T. harzianum* CBS 226.95. In contrast, only two mitogenomes, from *T. reesei* QM9414 and *T. atroviride* ATCC 26799, contained all positive values for both AT-/GC-skew (Table 1).

**Figure 3:**
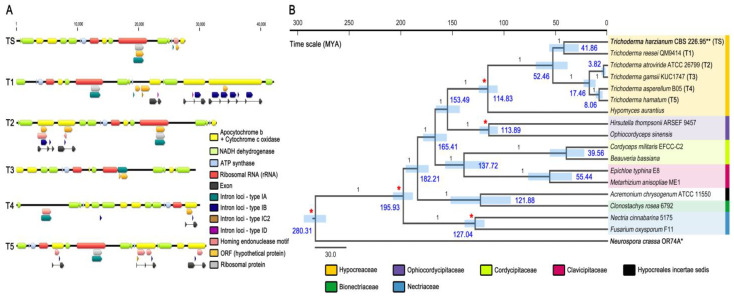
Comparative analysis of *Trichoderma* mitogenomes—I. (**A**) Comparison of the gene components. **TS**, *T. harzianum* CBS 226.95; **T1**, *T. reesei* QM9414; **T2**, *T. atroviride* ATCC 26799; **T3**, *T. gamsii* KUC1747; **T4**, *T. asperellum* B05; **T5**, *T. hamatum*. (**B**) Bayesian chronogram for the evolutionary divergences of *Trichoderma* mitogenomes. Estimated chronological times were presented in the time scale of Mya (million years ago), with 95% confidence interval values in blue bars. Bayesian posterior probabilities (BPPs) values and calibration points were indicated at nodes using numbers (black) and asterisks (red), respectively. Asterisks for the *Neurospora crassa* OR74A mitogenome (*) and *T. harzianum* CBS 226.95 mitogenome (**).

The mitogenome of *T. atroviride* ATCC 26799 was shown to harbor more protein CDS and *trn* genes than other *Trichoderma* species (Table 1). Across *Trichoderma* species, gene lengths for apocytochrome *b* (*cob*) and cytochrome *c* oxidases (*cox1*, *cox2*, and *cox3*) were observed in various sizes due to the presence of introns (Table S6). The *T. reesei* QM9414 mitogenome, which contains both the most extensive length of entire sequences and restricted proportions of non-coding regions, was observed to carrying more introns than other *Trichoderma* species. A total of 11 intron loci were detected, mainly in the exon–intron structures of the PCGs for apocytochrome *b* (*cob*) and cytochrome *c* oxidases (*cox1*, *cox2*, and *cox3*), which increase the full size of the target gene itself than the total number of annotated genes in the mitogenome sequences, whereas few introns were predicted in the *T. gamsii* KUC1747 and *T. harzianum* CBS 226.95 (Table 1 and Table S8). Notably, an IA-type intron (i.e., an intron of the subtype IA) was commonly found with an intronic ORF in the region of the *rrnL* gene of all *Trichoderma* species except *T. asperellum* B05 (i.e., in this study, the likelihood of the *rrnL* gene being present in the *T. asperellum* B05 mitogenome was not considered because this gene was not reported in the GenBank dataset of the *T. asperellum* B05 mitogenome) (Figure 3A and Table S8). Finally, compared with other *Trichoderma* species, the mitogenome of *T. harzianum* CBS 226.95 showed the highest identity of 95.17% to *T. reesei* QM9414 (Table 1).

### 3.4. Divergence Times of the Trichoderma Mitogenomes in Hypocreales

To explore the evolutionary divergence of *Trichoderma* mitogenomes in Hypocreales, a range for the taxonomic analysis was set to cover major families of the order of Hypocreales with species, such as (1) 10 selected species that represented each significant family in the order Hypocreales (one for Bionectriaceae (*Clonostachys rosea* 6792), two for Clavicipitaceae (*Epichloe typhina* E8 and *Metarhizium anisopliae* ME1), two for Cordycipitaceae (*Cordyceps militaris* EFCC-C2 and *Beauveria bassiana*), one for Hypocreales incertae sedis (*Acremonium chrysogenum* ATCC11550), two for Nectriaceae (*Nectria cinnabarina* 5175 and *Fusarium oxysporum* F11), and two for Ophiocordycipitaceae (*Hirsutella thompsonii* ARSEF9457 and *Ophiocordyceps sinensis*)), (2) one strain *H. aurantius* from the genus *Hypomyces*, to use as a sub-outgroup for the genus *Trichoderma* within the Hypocreaceae clade, and (3) one strain *N. crassa* OR74A, belonging to the order Sordariales that composed of the class Sordariomycetes with the order Hypocreales, to use as an outgroup for all Hypocreales species. Based on the complete mitogenomes of the selected species (Table S5), only exons sequences of the 13 core PCGs were used in the phylogenetic analysis to remove the potential of evolutionary effects by the presence/numbers of introns within the nucleotide sequences of the target gene.

On the time-scaled Bayesian phylogeny, all positions of species were well supported with high BPPs values (BPP = 1.00). Five calibration points were also presented with significant node ages, which were entirely consistent with those of the Bayesian chronogram previously reported using orthologous proteins of Hypocreales genomes (i.e., including *Trichoderma* genomes) [20].

Under the occurrence of the family Hypocreaceae at 114.83 Mya, a divergence time of the *Trichoderma* genus was estimated to be 52.46 Mya, and the speciation events that evolved within the genus *Trichoderma* appear to occur during the following periods: (1) 41.86 Mya between *T. harzianum* CBS 226.95 and *T. reesei* QM9414; (2) 8.06 Mya between *T. asperellum* B05 and *T. hamatum*; and (3) 3.82 Mya between *T. atroviride* ATCC 26799 and *T. gamsii* KUC1747. Interestingly, when considering the divergent times of *Trichoderma* species that dated on the Bayesian chronogram using orthologous of Hypocreales genomes (i.e., including *Trichoderma* genomes) [20], the speciation between *T. harzianum* CBS 226.95 and *T. reesei* QM9414 (41.86 Mya) was comparable to that of *Trichoderma* genomes (46 Mya; [20]) (Figure 3B). However, the speciation events of the *Trichoderma* clade consisting of four species (*T. asperellum* B05, *T. atroviride* ATCC 26799, *T. gamsii* KUC1747, and *T. hamatum*) evolved later (8.06–3.82 Mya) than those of *Trichoderma* genomes (25–11 Mya; [20]) (Figure 3B), which may be an indication of the mitogenome evolution that more recently and independently occurred apart from nuclear genomes.

## 4. Discussion

### 4.1. Putative tRNAs Overlapped with Protein-Coding Genes

Considering the tRNA genes that fully or partially overlapped with PCGs, some studies of metazoan/nematode mitogenomes have noted that it may allow (1) avoiding the loss of tRNA genes or (2) keeping gene functions by the co-evolution of two overlapping genes, on the mitogenome evolving to compacted lengths [56,59]. Recently, several putative *trn* genes, fully positioned within the PCGs of *Trichoderma* mitogenomes, have been described in our study of the *T. atroviride* ATCC 26799 mitogenome (e.g., *tRNA*^Val^ overlapped with the 3′ end region of *nad6* gene, *tRNA*^Met^ integrated within the 5′ end region of *nad2* gene) [11]. Similarly, a putative *trnV*^Val^ gene, fully overlapped with the 3′ end region of the *nad6* gene, was also found in the complete *de novo* mitogenome of *T. harzianum* CBS 226.95 (Tables S2 and S7).

The tRNA genes of mitochondria are expected to have evolved through various processes, such as (1) duplications or degenerations of tRNAs, (2) transpositions of tRNAs to the nucleus, causing the reduced tRNAs in the mitogenome itself, and (3) mutations of the tRNA anticodon, leading a remodeling of the tRNA secondary structure, however, the molecular-/functional aspects of these mitochondrial tRNAs have not yet been fully understood [60,61]. Interestingly, among the *Trichoderma* mitochondrial tRNAs, which are responsible for 20 standard amino acids in the translation system of mitochondrial coding proteins, a putative *trnV*^Val^ gene was only found for the amino acid valine across all *Trichoderma* species (Table S7). This feature requires further study concerning (1) the expression of putative *trnV*^Val^ as a form of functional tRNA, (2) whether it is co-expressed within the mature mRNA of PCGs, and (3) whether the mitochondria require a nucleus-encoded *trnV*^Val^ from the cytosol for the mitochondrial translation system (i.e., because the putative *trnV*^Val^, which is positioned within the protein coding sequences, might already be recognized as a pseudo-tRNA by the tRNA degenerations). Given these questions, all putative *trn* genes that were observed in the PCGs (*trnV*^Val^ and *tRNA*^Met^) actively support the likelihood of complex and dynamic evolutions of tRNAs in *Trichoderma* mitogenomes.

### 4.2. Evolution of Mitochondrial Introns in Trichoderma

In our recent study of the *T. atroviride* ATCC 26799 mitogenome [11], *Trichoderma* mitochondrial introns were characterized with (1) the similarities of intron sequences between *Trichoderma* species, though not clustered in most within the range of the *Trichoderma* genus, (2) potentials of the horizontal transfer of introns between inter-/intraspecific fungal diversities over the genus level of *Trichoderma*, and (3) several gain/loss events of introns on the reconstructed ancestral states of these introns [11]. In contrast to previous studies based on the most homologous introns of the core PCGs, in this study on the mitogenome of *T. harzianum* CBS 226.95, only two intron loci (all subtype IA) were observed regardless of the core PCGs: one was found in the *rrnL* gene with two internal ORFs (*orf408* and *rps3*) as an *rrnL-i* (i.e., intron placed within the *rrnL* gene), another was also detected with multiple intronic ORFs that were composed by embedding the *orf170* gene in an 864 bp-sized HE harboring a GIY-YIG motif (Figure 3A, Tables S1 and S8). Nevertheless, compared among six *Trichoderma* species, all features of the *Trichoderma* mitochondrial introns that were described in our previous study are still meaningful through the constant patterns of intron distributions in *Trichoderma* species (e.g., most of the intron loci were detected in the exon–intron structures of PCGs for apocytochrome *b* (*cob*) and cytochrome *c* oxidases (*cox1*, *cox2*, and *cox3*), but not in the two species of *T. gamsii* KUC1747 and *T. harzianum* CBS 226.95 that harbor all core PCGs on the intronless structures) (Figure 3A and Table S8).

Since mitochondrial endosymbiosis occurred between the earliest ancestor of mitochondria (pre-mitochondrial alphaproteobacterium) and an archaeal-derived host (Asgard archaea), the ancestral mitochondria genome had experienced massively reduced mitogenomes, similar to modern mitochondria [1]. However, despite its ultimately compact size that was driven by reductive evolutionary processes (e.g., mainly, the loss of redundant/unnecessary genes and/or endosymbiotic gene transfers to the nucleus) [1], the structural intricacy of the current mitogenome varies immensely across eukaryotes [4,62]. In fungal mitogenomes, the group I intron, which is capable of self-splicing, causes the architectural complex of the mitogenome [32,63,64]. Among six *Trichoderma* mitogenomes, the existence of mitochondrial introns was involved in the structural complexity of the mitogenome itself (Figure 3A), however, strictly not leading to the increase in the entire lengths of their mitochondrial sequences. Excluding *T. reesei* QM9414, a correlation between the numbers of introns present and the increase in mitogenome size could not be typically defined across *Trichoderma* species, suggesting the significance of the location and size of the intron locus rather than the intron’s existence itself within mitogenome sequences (Table 1 and Table S8).

Including our previous study of the *T. atroviride* ATCC 26799 mitogenome [11], several recent studies of fungal species have demonstrated the evolutionary dynamics of mitochondrial introns, such as multiple-gains/losses/degenerations of introns, by tracing the ancestral history of these intron loci within the relevant diversity of the target fungus [9,65]. In this study, ancestral states of intron events across all six *Trichoderma* species were not constructed due to the absence of homologous intron loci of the core PCGs within the *T. harzianum* CBS 226.95 mitogenome (Figure 3A and Table S8). However, when comparing phylogenies, six *Trichoderma* mitogenomes were identically arranged in two kinds of BI topologies, which were obtained either by using full genes (including introns) of the core PCGs (Figure 2) or by only using exon sequences (excluded introns) of the core PCGs (Figure 3B), such as (1) a clade by *T. atroviride* ATCC 26799 and *T. gamsii* KUC1747, (2) a clade by *T. asperellum* B05 and *T. hamatum*, and (3) a clade by *T. harzianum* CBS 226.95 and *T. reesei* QM9414. In particular, the clustering between *T. reesei* QM9414 (the largest sized one with the highest number of intron loci) and *T. harzianum* CBS 226.95 (the smallest lengths harboring few introns) deserved attention related to the divergence time of this clade that was estimated to be much older (~41.86 Mya) than the other two clades (8.06–3.82 Mya) (Figure 2 and Figure 3B). Given the theory of genomic streamlining [66], some, but not all, species of lichenized fungi are speculated to have experienced multiple intron loss events for mitogenome contractions [10]. However, in the evolutionary view of eukaryogenesis, the origin and evolution of introns have been considered using the comprehensive perspectives of two competing theories: the ‘introns-early’ theory (i.e., introns first existed at the earliest stages of life’s evolution, then evolved toward intron loss) and the ‘introns-late’ theory (i.e., introns emerged/increased in eukaryotes during eukaryote evolution)’ [67]. All *Trichoderma* clustering discovered in this study seems to be insufficient to infer whether the *Trichoderma* mitogenomes evolved to reduce mitogenome sizes due to intron loss and degeneration under genome streamlining, but clearly imply the continuation of varied-gain and loss of mitochondrial introns during *Trichoderma* evolution (Figure 2 and Figure 3B).

When considering the identical arrangements of *Trichoderma* clustering between two BI topologies that were obtained with/without intron sequences of the core PCGs (Figure 2 and Figure 3B), it actively supports a shared evolutionary history between the core PCGs and their introns in *Trichoderma*. Meanwhile, IA-typed *rrnL-i* was commonly detected across all *Trichoderma* species except *T. asperellum* B05 (i.e., in this study, the likelihood of the *rrnL* gene being present in the *T. asperellum* B05 mitogenome was not considered because this gene was not reported in the GenBank dataset of the *T. asperellum* B05 mitogenome (GenBank accession no. NC_037075)) (Figure 3A and Table S8). Although it should be comprehensively reflected in the unique features of other intron loci, such as (1) the intron locus harboring multiple intronic ORFs, or (2) the coexistence of different intron subtypes within a single coding gene (Table S8); nevertheless, the *rrnL-i* is expected to play an essential role in revealing the dynamic history of *Trichoderma* mitochondrial introns, as one of the primitive factors that is assumed to be inherited from the most recent common ancestor of the *Trichoderma* genus.

### 4.3. Time-Scaled Bayesian Phylogeny with the Strain T. harzianum HB324

**Figure 4:**
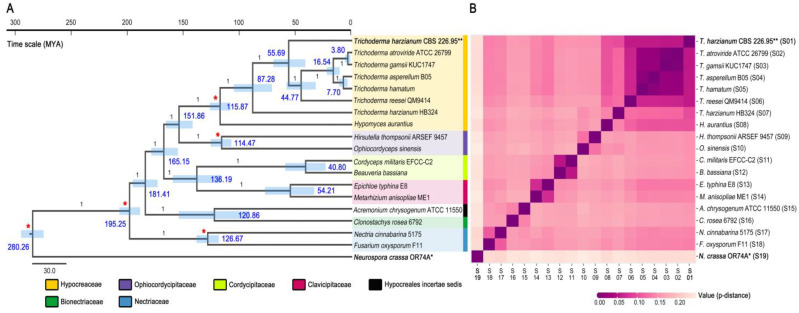
Comparative analysis of *Trichoderma* mitogenomes—II. (**A**) Time-scaled Bayesian tree on *Trichoderma* mitogenomes that includes a new isolate of *Trichoderma*. Estimated chronological times were presented in time scale in the time scale of Mya (million years ago), with 95% confidence interval values in blue bars. Bayesian posterior probabilities (BPPs) values and calibration points were indicated at nodes using numbers (black) and asterisks (red), respectively. (**B**) Pairwise genetic distance among Hypocreales mitogenomes. All used fungal species (including *N. crassa* OR74A as an outgroup) and obtained p-distance values are described in Tables S5 and S10, respectively. Asterisks for the *Neurospora crassa* OR74A mitogenome (*) and *T. harzianum* CBS 226.95 mitogenome (**).

In addition to the six complete mitogenomes of *Trichoderma* species (including *T. harzianum* CBS 226.95 of this study), there is additional mitogenomic information from the group of *T. harzianum* species; the 32,277 bp mitogenome of *T. harzianum* HB324 (GenBank accession no. MT263519). Nonetheless, defined annotations of this mitogenome are unavailable in GenBank because it was publicly deposited as an unverified dataset (as of January 2021). In a recent study of this mitogenome that described mitogenomic features for the first time [68], some coding genes were suggested with multiple terminations of their coding regions. For these reasons, in this study, the core PCGs of the *T. harzianum* HB324 mitogenome were re-annotated to remove the positions of stop codons that were multiply predicted in the middle of coding genes and re-defined their gene lengths (Table S9). Then, the evolutionary divergences of *Trichoderma* mitogenomes were re-traced with *T. harzianum* HB324 harboring newly modified annotations, under the same analytical conditions that applied for the BI phylogeny in Figure 3B. On the newly constructed time-scaled Bayesian tree, all fungal species were well-positioned with high BPPs values (BPP = 1.00), and five calibration points were also indicated to be compatible with those of the Bayesian chronogram using orthologous of Hypocreales genomes (i.e., including *Trichoderma* genomes) [20]. However, despite belonging to the same species of *T. harzianum*, two strains of CBS 226.95 and HB324 were placed separately (Figure 4A).

As shown in the two BI topologies (Figure 2 and Figure 3B), phylogenetic replacements of the complete six *Trichoderma* mitogenomes (i.e., *T. asperellum* B05, *T. atroviride* ATCC 26799, *T. gamsii* KUC1747, *T. hamatum*, *T. harzianum* CBS 226.95, and *T. reesei* QM9414) were congruent with those of the chronogram using 638 core orthologous from Hypocreales genome [20], such as (1) a clade by *T. harzianum* and *T. reesei*, and (2) a clade consisting of subclades by four *Trichoderma* species (*T. asperellum*, *T. atroviride*, *T. gamsii*, and *T. hamatum*). However, as shown in Figure 4A, among the seven *Trichoderma* mitogenomes (including strain HB324), *T. harzianum* HB324 was positioned as an outgroup to all *Trichoderma* taxa. At the same time, *T. harzianum* CBS 226.95 was clustered with a subclade comprised of the five remaining *Trichoderma* species. According to the joining of strain HB324, *Trichoderma* speciation was also predicted more divergently. The divergence of *T. harzianum* HB324 was estimated at 87.28 Mya and followed by *T. harzianum* CBS 226.95 at 55.69 Mya. Furthermore, *T. reesei* QM9414 appeared later than *T. harzianum* CBS 226.95 at 44.77 Mya (Figure 4A).

Compared to *T. harzianum* CBS 226.95, the mitogenome of *T. harzianum* HB324 was approximately 4645 bp larger and exhibited a sequence identity of 92.23% (query coverage 94%). The GC content was similar, but skewness patterns were different for all positive values in *T. harzianum* HB324. As described in the beginning of this discussion, putative *trn* genes entirely positioned within the coding genes (e.g., *tRNA*^Val^ of the *nad6* gene, *tRNA*^Met^ of the *nad2* gene) were found across all *Trichoderma* species (Table S7)—however, not in the *T. harzianum* HB324 (Table S9). In addition, when comparing genetic distances using exon sequences of the 13 core PCGs (*atp6*, *atp8*, *cob*, *cox1*, *cox2*, *cox3*, *nad1*, *nad2*, *nad3*, *nad4*, *nad4L*, *nad5*, and *nad6*; the *atp9* gene was excluded because it has not been reported in the GenBank dataset of the *T. gamsii* KUC1747 mitogenome (GenBank accession no. KU687109)), *T. harzianum* HB324 was assumed to be more divergent from *T. harzianum* CBS 226.95 (p-distance value, 0.099) than other *Trichoderma* species (p-distance values, 0.055–0.057) (Figure 4B and Table S10). All these aspects showed a presence of mitogenomic differentiation between two *T. harzianum* strains, CBS 226.95 and HB324. Nevertheless, it seems that one additional point should be also contemplated together as below.

Since the first description of the name *Trichoderma* [69], phylogenetic concepts of the genus *Trichoderma* have been developed to systematically identify *Trichoderma* taxonomy [70]. Particularly, in earlier studies, some *Trichoderma* strains were misidentified, and even the name of *T. harzianum* has been misused for many different species due to the species complex of *Trichoderma* [71,72,73,74]. To overcome these difficulties, species of *Trichoderma* have recently been (re-)identified for the most accurate discrimination between *Trichoderma* using combined approaches, such as (1) morphological phenotypes and (2) simultaneous applications using multiple-DNA barcodes on regions of rDNA internal transcribed spacers (ITS), elongation factor 1-α (tef1), and RNA polymerase II subunit (RPB2) [74,75]; consequentially, a taxonomy of the *T. harzianum* species complex was revised to include 14 species [74]. For instance, the strain P1 (ATCC 74058), strain T22, and strain ATCC 26799, widely known as representatives of *T. harzianum* species in earlier studies, were re-determined to be *T. atroviride* P1 [71], *T. afroharzianum* T22 [74], and *T. atroviride* ATCC 26799 [75], respectively. Meanwhile, the fungus *T. harzianum* HB324, isolated from the leaves of rubber tree (*Hevea brasiliensis*), was reported to be identified using a DNA barcode only for the ITS region (used PCR primers for ITS4 and ITS5) [76].

In a recent study of the *T. harzianum* HB324 mitogenome [68], a time-scaled maximum likelihood (ML) tree was constructed using only three coding genes (*atp8*, *atp9*, and *cox3*). On this ML tree, the mitogenome of *T. harzianum* HB324 occurred at 27.40 Mya, placed apart from the clade covering all other *Trichoderma* species [68]. In contrast, though disagreements of analysis conditions must be considered (e.g., tree platform, applied gene sets), the clustering of *Trichoderma* species on the time-scaled BI tree obtained in this study was congruent with those of the chronogram using nucleus orthologous proteins of Hypocreales [20] (Figure 3B). Nevertheless, when considering the newly constructed BI chronogram by including strain HB324 (Figure 4A), it is evident that the mitogenome of *T. harzianum* HB324 has distinctive features, which influenced the *Trichoderma* divergences dynamically. Taxonomic revisions of *Trichoderma* species are still ongoing to correctly identify members of the *T. harzianum* complex [77]. Obtaining improved resolutions of *Trichoderma* topologies, including new isolates such as the strain HB324, will be more useful for the elucidation of *Trichoderma* mitogenome evolutionary relationships.

### 4.4. Evolutionary Pressures for the Trichoderma Mitogenomes

Related with the adaptive evolution of *Trichoderma* mitogenomes, selective pressures were investigated using the codon alignments of 13 core PCGs (*atp6*, *atp8*, *cob*, *cox1*, *cox2*, *cox3*, *nad1*, *nad2*, *nad3*, *nad4*, *nad4L*, *nad5*, and *nad6*; the *atp9* gene was excluded because it has not been reported in the GenBank dataset of the *T. gamsii* KUC1747 mitogenome (GenBank accession no. KU687109)). Compared to the Ka/Ks ratios, most of the core PCGs were assessed with values lower than one (<1), which is supposed to be under purifying selection. However, interestingly, the *cox3* gene was measured with a value greater than one (1>) in a pairwise comparison between *T. harzianum* CBS 226.95 and *T. reesei* QM9414, implying a positive selection potential. When considering the *Trichoderma* clustering of the two BI topologies (Figure 2 and Figure 3B), the *cox3* gene might be a site for adaptive changes that influenced the evolutionary speciation events between two strains, CBS 226.95 and QM9414, which were closely placed in a sister relationship within the same clade.

**Figure 5:**
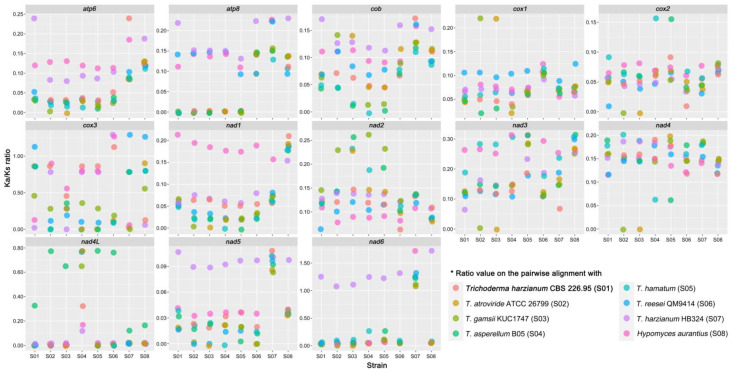
Analysis of Ka/Ks ratios of the core PCGs. Obtained values are described in Table S11. Ka, nonsynonymous substitution rate; Ks, synonymous substitution rate.

Another remarkable thing is that the *nad6* gene of *T. harzianum* HB324 was shown with a Ka/Ks ratio over one (1>) against those of all other *Trichoderma* species, even including *H. aurantius* belonging to the genus *Hypomyces* that consisted of the family Hypocreaceae. It seems to support the potential of evolutionarily diversifying for the fungus HB324, apart from the other species within the clade of all *Trichoderma* taxa as shown in Figure 4A (Figure 5 and Table S11). Meanwhile, using the MEME approach on the phylogenetic lineages of Hypocreales (Figure 4A), the *cox1* gene was predicted with higher individuals of 20 codon sites that were assumed to have evolved under a positive diversifying selection, in contrast to the non-sites in the *atp8* gene (Table S12).

Under intense selective pressures, organisms may evolve adaptations for their survival in certain ecological conditions, which would be achieved via site-specific substitutions of nucleotides/amino acids affecting the protein structure, further leading to changes in protein functions [78]. In a previous study of the evolution of *Trichoderma* habitat preferences, *T. reesei* was found to favor decaying plant materials rather than soil [14]. The OXPHOS pathway is strictly dependent on oxygen to generate cellular energy [79]. When considering the various habitats where *Trichoderma* have been isolated (e.g., soils, wood, and living plants/fungi) [14], environmental conditions, particularly concerning oxygen, might have affected the evolution of *Trichoderma* mitogenomes during their adaptive diversification. Our results can be proposed as clear signals of the evolving effects on the essential genes of OXPHOS, further expanding the viewpoint of molecular- and metabolic evolutions of *Trichoderma* mitochondria under the inherited habits of their geographic origins.

## 5. Conclusions

Here, a complete *de novo* mitogenome of an ex-neotype fungus of the *T. harzianum* species complex, *T. harzianum* CBS 226.95, is presented for the first time. *Trichoderma* mitogenomes were comprehensively compared to update the genomic understanding of *Trichoderma* mitochondria, the evolutionary divergences of *Trichoderma* mitogenomes were also exclusively traced using the mitochondrial core PCGs and discussed with latent genomic features triggered mitogenomic evolution in *Trichoderma*. Our results demonstrate that the adaptive evolution of *Trichoderma* mitogenomes has been actively ongoing with regard to the complex effects of various genetic elements. All of this information will allow for a better understanding of the distinct behaviors of *Trichoderma* species in nature, thus further expanding our insight into *Trichoderma* to make them more effective fungal biocontrol agents.

## Figures and Tables

**Table 1:** Comparison of mitochondrial genomic features among *Trichoderma* species.

Strain	*T. harzianum* CBS 226.95 ^a^	*T. reesei* QM9414 ^a^	*T. atroviride* ATCC 26799 ^a^	*T. gamsii* KUC1747 ^a^	*T. asperellum* B05 ^a^	*T. hamatum* ^a^
GenBank accession no.	MN564945	AF447590	MN125601	KU687109	NC_037075	MF287973
Mitochondrial genome size (bp)	27,632	42,130	32,758	29,303	29,999	32,763
AT content (%)/ GC content (%)	72.5/27.6	72.8/27.2	71.8/28.2	71.7/28.3	72.2/27.8	72.3/27.7
AT-skew/ GC-skew	(−)0.004/0.098	0.041/0.086	0.006/0.091	(−)0.062/0.036	(−)0.066/0.043	(−)0.002/0.031
Protein CDS/ tRNAs ^b^/rRNAs	18/24 (1)/2	19/23 (2)/2	21/27 (1)/2	18/26 (1)/2	17/25 (1)/1	20/26 (1)/2
Intergenic region (%)	20.75	17.58	24.93	30.15	31.73	19.05
Genome identity (%) (query coverage)	Used as query	95.17 (93.0%)	95.71 (88.0%)	95.52 (88.0%)	94.56 (88.0%)	94.69 (87.0%)
No. of intron locus ^c^ (subtype)	2 (IA (2))	11 (IA (2), IB (6), C2 (1), ID (2))	4 (IA (1), IB (3))	1 (IA (1))	3 (IA (1), IB (2))	5 (IA (1), IB (3), IC2 (1))
Note	This study	-	-	Non-existence of *atp9* gene	Non-existence of *rrnL* gene	-
In Figure 3A, indicated as	TS	T1	T2	T3	T4	T5

^a^ Based on the GenBank database, NCBI (datasets as of, January 2021). ^b^ A number of putative *trn* genes predicted within the protein-coding genes were indicated inside the parentheses (details in Table S7). ^c^ Details of the detected group I intron loci (subtype, position, intronic ORF) are shown in Table S8.

## Data Availability

The complete *de novo* mitochondrial genome sequence of *Trichoderma harzianum* CBS 226.95 is openly available in GenBank (accession number MN564945).

## Supplementary Materials

The following are available online at https://www.mdpi.com/article/10.3390/microorganisms9081564/s1, Figure S1. Predicted secondary structure of transfer RNA genes in the *T. harzianum* CBS 226.95 mitogenome.; Figure S2. Phylogenetic tree of the Hypocreales species (Sordariomycetes) mitochondrial genomes based on ML (maximum likelihood) analysis; Table S1. Genomic organization of the *T. harzianum* CBS 226.95 mitogenome; Table S2. Transfer RNA genes identified in the mitogenome of *T. harzianum* CBS 226.95; Table S3. Codon usage in the mitogenome of *T. harzianum* CBS 226.95; Table S4. Identification of repetitive elements in the mitogenome of *T. harzianum* CBS 226.95; Table S5. All Sordariomycetes mitogenomes that are used for phylogenetic analyses in this study; Table S6. Gene components for the core-PCGs and rRNAs in the *Trichoderma* complete mitogenomes; Table S7. Putative tRNA gene (*trn*) within the protein-coding gene of *Trichoderma* mitogenomes; Table S8. Positions of detected group I intron loci on the mitogenomes of *Trichoderma* species; Table S9. Comparison of the mitochondrial genomic features between *T. harzianum* CBS 226.95 and *T. harzianum* HB324; Table S10. Estimated p-distance values among the Hypocreales mitogenomes; Table S11. Ka/Ks ratios for the 13-core protein-coding genes in the Hypocreaceae mitogenomes; Table S12. Prediction of codon sites evolving under positive diversifying selection on the MEME approach.
