# Supplementary material for: An Update on Trichoderma Mitogenomes: Complete De Novo Mitochondrial Genome of the Fungal Biocontrol Agent Trichoderma harzianum (Hypocreales, Sordariomycetes), an Ex-Neotype Strain CBS 226.95, and Tracing the Evolutionary Divergences of Mitogenomes in Trichoderma
Source: Microorganisms. 2021 Jul 23;9(8):1564. doi: 10.3390/microorganisms9081564 (PMC8401334; doi:10.3390/microorganisms9081564)
Supplement: Supplementary file 1 [file microorganisms-09-01564-s001.zip › microorganisms-1285849-supplementary/Supplementary_Figure_Legend.pdf]

SUPPLEMENTARY FIGURE LEGENDS

Figure S1. Predicted secondary structure of transfer RNA genes (*trn* genes) that identified in the *T. harzianum* CBS 226.95 mitogenome. All tRNA genes were indicated with numbers corresponding nucleotide positions in the mitogenome. A putative $trnV^{\text{Val}}$ gene that predicted fully within the coding region of the *nad6* gene was indicated by an asterisk.

Figure S2. Phylogenetic tree of the Hypocreales species (Sordariomycetes) mitochondrial genomes based on ML (maximum likelihood) analysis. The tree was generated using concatenated sequences of 13 core genes (*atp6, atp8, cob, cox1*, *cox2*, *cox3*, *nad1*, *nad2*, *nad3*, *nad4*, *nad4L*, *nad5*, and *nad6*), and the mitochondrial genome of *Neurospora crassa* OR74A (Sordariales) was used as an outgroup. All Sordariomycetes species that used for the phylogenetic tree were described in **Table S5**, and ML bootstrap (BS) values were marked on the nodes.
