# Supplementary material for: An Update on Trichoderma Mitogenomes: Complete De Novo Mitochondrial Genome of the Fungal Biocontrol Agent Trichoderma harzianum (Hypocreales, Sordariomycetes), an Ex-Neotype Strain CBS 226.95, and Tracing the Evolutionary Divergences of Mitogenomes in Trichoderma
Source: Microorganisms. 2021 Jul 23;9(8):1564. doi: 10.3390/microorganisms9081564 (PMC8401334; doi:10.3390/microorganisms9081564)
Supplement: Supplementary file 1 [file microorganisms-09-01564-s001.zip › microorganisms-1285849-supplementary/Supplementary_Tables.pdf]

Table S1. Genomic organization of the *T. harzianum* CBS 226.95 mitogenome.

Gene	Strand[a]	Start position	Stop position	Length (bp)	Start codon	Stop codon	Note
nad4L	F	628	897	270	ATG	TAA	
nad5	F	897	2888	1992	ATG	TAA	overlap (-1) with *nad4L*
cob	F	3254	4423	1170	ATG	TAA	
cox1	F	5102	6688	1587	ATG	TAA	
nad1	F	7194	8303	1110	ATG	TAA	
nad4	F	8565	9941	1377	GTG	TAA	
atp8	F	10430	10576	147	ATG	TAA	
atp6	F	10695	11477	783	ATG	TAA	
rns	F	12055	13557	1503	-	-	small subunit rRNA
cox3	F	14058	14867	810	ATG	TAA	
nad6	F	15111	15863	753	ATG	TAA	putative *trnV*^{Val}
rrnL	F	16743	21455	4713	-	-	large subunit rRNA
rps3	F	19425	20792	1368	ATA	TAA	ribosomal protein S3
orf408	F	19566	20792	1227	ATG	TAA	hypothetical protein
nad2	F	23140	24807	1668	ATG	TAA	
nad3	F	24808	25221	414	ATG	TAA	
atp9	F	25409	25603	195	ATG	TAG	
orf170	F	26144	26656	513	ATG	TAG	hypothetical protein
cox2	F	26883	27632	750	ATG	TAA	
Homing endonuclease	F	25793	26656	864	ATA	TAG	GIY-YIG

Other features	Value
Mitochondrial genome size (bp)	27,632
AT content (%) / GC content (%)	72.45 (A, 36.1; T, 36.4) / 27.55 (G, 15.1; C, 12.4)
AT-skew / GC-skew	(-)0.004 / 0.098
Intergenic region (%)[b]	20.75
No. of tRNAs (%)[b]	24 (6.49)
No. of rRNAs (%)[b]	2 (22.50)
GenBank accession no.	**MN564945**

[a]F, forward strand.

[b]% of total nucleotide sequences (nt) of the mitogenome.

Table S2. Transfer RNA genes that identified in the mitogenome of *T. harzianum* CBS 226.95.

Gene (product)	Strand[a]	Start position	Stop position	Lenth (nt)	Anticodon
trnR (trnR-Arg)	F	85	155	71	ACG
trnC (trnC-Cys)	F	4536	4607	72	GCA
trnR (trnR-Arg)	F	6786	6856	71	TCT
trnY (trnY-Tyr)	F	13598	13681	84	GTA
trnD (trnD-Asp)	F	13775	13848	74	GTC
trnS (trnS-Ser)	F	13854	13937	84	GCT
trnN (trnN-Asn)	F	13942	14013	72	GTT
trnG (trnG-Gly)	F	14930	15000	71	TCC
trnI (trnI-Ile)	F	16214	16285	72	GAT
trnS (trnS-Ser)	F	16306	16392	87	TGA
trnW (trnW-Trp)	F	16401	16472	72	TCA
trnP (trnP-Pro)	F	16526	16597	72	TGG
trnT (trnT-Thr)	F	21460	21530	71	TGT
trnE (trnE-Glu)	F	21536	21608	73	TTC
trnM (trnM-Met)	F	21609	21679	71	CAT
trnM (trnM-Met)	F	21834	21906	73	CAT
trnL (trnL-Leu)	F	21911	21993	83	TAA
trnA (trnA-Ala)	F	22079	22150	72	TGC
trnF (trnF-Phe)	F	22156	22228	73	GAA
trnK (trnK-Lys)	F	22321	22393	73	TTT
trnL (trnL-Leu)	F	22462	22545	84	TAG
trnQ (trnQ-Gln)	F	22623	22695	73	TTG
trnH (trnH-His)	F	22814	22886	73	GTG
trnM (trnM-Met)	F	22983	23054	72	CAT
trnV (trnV-Val)[b]	F	15781	15852	72	TAC

[a]F, forward strand.

[b]A putative *trn* gene predicted within the coding region of the *nad6* gene.

Table S3. Codon usage in the mitogenome of *T. harzianum* CBS 226.95.

Amino acid (AA)		Codon (% of AA)	Frequency value (%)[a]	Amino acid (AA)		Codon (% of AA)	Frequency value (%)[a]
A	Ala	GCA (30.16)	5.38	N	Asn	AAC (20.96)	6.23
		GCC (9.84)				AAT (79.04)	
		GCG (3.61)		P	Pro	CCA (24.10)	2.93
		GCT (56.39)				CCC (0.60)	
C	Cys	TGC (19.23)	0.46			CCG (3.01)	
		TGT (80.77)				CCT (72.29)	
D	Asp	GAC (14.09)	2.63	Q	Gln	CAA (98.20)	1.96
		GAT (85.91)				CAG (1.80)	
E	Glu	GAA (90.80)	3.07	R	Arg	AGA (81.33)	2.93
		GAG (9.20)				AGG (3.01)	
F	Phe	TTC (46.32)	7.20			CGT (15.66)	
		TTT (53.68)				CGA, CGC, CGG (0.00)	
G	Gly	GGA (48.56)	6.14	S	Ser	AGC (4.47)	8.30
		GGC (0.29)				AGT (41.49)	
		GGG (2.01)				TCA (20.21)	
		GGT (49.14)				TCC (1.49)	
H	His	CAC (27.78)	1.91			TCT (32.34)	
		CAT (72.22)				TCG (0.00)	
I	Ile	ATA (63.84)	11.22	T	Thr	ACA (47.96)	5.19
		ATC (6.92)				ACC (2.38)	
		ATT (29.25)				ACG (0.68)	
K	Lys	AAA (95.12)	5.07			ACT (48.98)	
		AAG (4.88)		V	Val	GTA (57.22)	6.48
L	Leu	CTA (5.98)	13.57			GTC (1.36)	
		CTC (0.13)				GTG (3.81)	
		CTG (0.52)				GTT (37.60)	
		CTT (9.10)		W	Trp	TGA (100)	1.29
		TTA (80.88)				TGG (0.00)	
		TTG (3.38)		Y	Tyr	TAC (29.43)	5.28
M	Met	ATA (1.44)	2.45			TAT (70.57)	
		ATG (97.84)		STOP	TER	TAA (83.33)	0.32
		GTG (0.72)				TAG (16.67)	

[a]Relative percentage (%) of total frequencies of expected codons.

Table S4. Identification of repetitive elements in the mitogenome of *T. harzianum* CBS 226.95.

Interspersed repeat sequences detected by BLAST searches of the *T. harzianum* CBS 226.95 mitogenome against itself (self-comparisons)

Matching no.	Identities (%)	E-value	Gaps	Repeat element position as a Query (Start position - Strop position)	Repeat element position as a Subject (Start position - Strop position)
1	79	3e-49	0	10,129 - 10,348	9,724 - 9,943
2	100	5e-21	0	22,741 - 22,793	22,636 - 22,688
3	73	9e-12	10	25,611 - 25,742	19,202 - 19,331

Tandem repeat sequences detected in the mitogenome of *T. harzianum* CBS 226.95

Matching no.	Indices[a]	Consensus size[b]	Copy number[c]	Matches / Mismatches / Indels (%)[d]	Consensus repeat pattern
1	698 - 736	20	1.9	85.0 / 0.0 / 15.0	TAATGCTATTATCTATAAAA
2	16,475 - 16,499	13	1.9	100.0 / 0.0 / 0.0	ATAATTATTAATT
3	19,780 - 19,829	14	3.8	87.0 / 5.0 / 8.0	AAAAAATAAATATT
4	19,771 - 19,829	26	2.2	82.0 / 12.0 / 6.0	TAAATAGAGAAAAAAAAATAAAAAAA
5	21,685 - 21,743	19	3.1	95.0 / 5.0 / 0.0	TTAATATAAATATATTAAA
6	21,691 - 21,742	9	5.6	79.0 / 4.0 / 17.0	TAAATATAT

[a]Indices of the repeat in the mitogenomic sequences.

[b]Size of the consensus repeat pattern.

[c]Number of copies that aligned with the consensus repeat pattern.

[d]Percent of Matches/Mismatches/Indels between adjacent copies of consensus repeat pattern.

Table S5. All Sordariomycetes mitogenomes that used for phylogenetic analyses in this study.

Species name[a]	Phylum (order : family)	Mitochondrial genome size (Kb)	GC (%)	Number of gene (total gene : protein CDS : rRNA : tRNA)[b]	**GenBank accession no.**
Acremonium chrysogenum ATCC 11550	Hypocreales : Hypocreales incertae sedis	27.27	26.5	45 : 17 : 2 : 26	**KF757229.1**
Acremonium fuci 3a34	Hypocreales : Hypocreales incertae sedis	24.57	28.8	45 : 15 : 2 : 28	**KR864757.1**
Akanthomyces muscarius (*Lecanicillium muscarium*)	Hypocreales : Cordycipitaceae	24.50	27.1	42 : 15 : 2 : 25	**AF487277.1**
Beauveria bassiana	Hypocreales : Cordycipitaceae	29.96	27.2	42 : 15 : 2 : 25	**EU371503.2**
Beauveria brongniartii (*Cordyceps brongniartii*)	Hypocreales : Cordycipitaceae	33.93	27.3	42 : 20 : 2 : 25	**EU100743.1**
Beauveria caledonica	Hypocreales : Cordycipitaceae	38.32	26.3	54 : 27 : 1 : 26	**KT201150.1**
Beauveria malawiensis	Hypocreales : Cordycipitaceae	44.14	26.7	56 : 30 : 1 : 25	**KT201147.1**
Beauveria pseudobassiana C1010	Hypocreales : Cordycipitaceae	28.01	27.5	43 : 15 : 3 : 25	**KF297618.1**
Calonectria ilicicola FJLY41	Hypocreales : Nectriaceae	39.89	28.5	48 : 20 : 2 : 26	**MT118655.1**
Clonostachys rosea 6792	Hypocreales : Bionectriaceae	40.92	27.9	42 : 15 : 2 : 25	**KU668563.1**
Cordyceps cicadae CCAD02	Hypocreales : Cordycipitaceae	56.58	26.1	70 : 43 : 2 : 25	**MH922223.1**
Cordyceps militaris EFCC-C2	Hypocreales : Cordycipitaceae	33.28	26.8	43 : 15 : 2 : 26	**KF432176.1**
Epichloe festucae AR5	Hypocreales : Clavicipitaceae	88.74	27.5	85 : 60 : - : 25	**KX066186.1**
Epichloe typhina E8	Hypocreales : Clavicipitaceae	84.63	27.0	81 : 56 : - : 25	**KX066185.1**
Fusarium bambusae 5137	Hypocreales : Nectriaceae	63.59	31.9	44 : 15 : 2 : 27	**MH684411.1**
Fusarium cerealis	Hypocreales : Nectriaceae	93.16	31.7	82 : 52 : 2 : 28	**MT036639.1**
Fusarium circinatum MRC 7870	Hypocreales : Nectriaceae	67.11	31.4	44 : 30 : 2 : 27	**JX910419.1**

Fusarium commune JCM11502	Hypocreales : Nectriaceae	47.53	32.4	46 : 18 : 2 : 26	**NC_036106.1**
Fusarium culmorum CBS 139512	Hypocreales : Nectriaceae	103.84	31.7	90 : 60 : 2 : 28	**KP827647.1**
Fusarium gerlachii CBS 123666	Hypocreales : Nectriaceae	93.43	31.9	83 : 53 : 2 : 28	**KM486533.1**
Fusarium mangiferae	Hypocreales : Nectriaceae	30.63	31.3	28 : 18 : 1 : 9	**KP742838.1**
Fusarium oxysporum F11	Hypocreales : Nectriaceae	34.48	31.0	17 : 16 : 2 : 25	**AY945289.1**
Fusarium oxysporum UASWS AC1	Hypocreales : Nectriaceae	51.54	31.9	49 : 22 : 1 : 26	**KR952337.1**
Fusarium pseudograminearum	Hypocreales : Nectriaceae	110.53	31.6	95 : 65 : 2 : 28	**MT036635.1**
Fusarium solani mpVI	Hypocreales : Nectriaceae	62.98	28.9	57 : 30 : 2 : 25	**JN041209.1**
Fusarium verticillioides (*Gibberella moniliformis*) 7600	Hypocreales : Nectriaceae	53.75	32.6	50 : 21 : 2 : 27	**NC_016687.1**
Hirsutella minnesotensis 3608	Hypocreales : Ophiocordycipitaceae	52.25	28.4	56 : 30 : 2 : 25	**KR139916.1**
Hirsutella rhossiliensis	Hypocreales : Ophiocordycipitaceae	62.48	28.2	47 : 24 : 2 : 26	**KU203675.1**
Hirsutella thompsonii ARSEF 9457	Hypocreales : Ophiocordycipitaceae	62.51	29.8	59 : 30 : 2 : 27	**MH367294.1**
Hirsutella vermicola	Hypocreales : Ophiocordycipitaceae	53.79	25.3	54 : 27 : 2 : 25	**KY465721.1**
Hypomyces aurantius	Hypocreales : Hypocreaceae	71.64	28.3	71 : 44 : 2 : 25	**KU666552.1**
Ilyonectria destructans	Hypocreales : Nectriaceae	42.90	28.2	46 : 15 : 2 : 29	**KU881725.1**
Lecanicillium saksenae	Hypocreales : Cordycipitaceae	25.92	26.5	43 : 15 : 2 : 26	**KT585676.1**
Metarhizium anisopliae ME1	Hypocreales : Clavicipitaceae	24.67	28.4	41 : 15 : 2 : 24	**AY884128.1**
Nectria cinnabarina 5175	Hypocreales : Nectriaceae	69.90	28.7	42 : 15 : 2 : 25	**KT731105.1**
Ophiocordyceps sinensis	Hypocreales : Ophiocordycipitaceae	157.54	30.2	117 : 88 : 2 : 27	**KY622006.1**
Paecilomyces penicillatus SAAS_ppe1	Hypocreales : Clavicipitaceae	27.48	27.5	43 : 17 : 2 : 24	**MK069583.1**
Parengyodontium album	Hypocreales : Cordycipitaceae	28.08	25.9	43 : 17 : 2 : 24	**KX061492.1**

Pochonia chlamydosporia (*Metacordyceps chlamydosporia*) 170	Hypocreales : Clavicipitaceae	25.62	28.3	39 : 15 : 2 : 22	**KF479445.1**
Tolypocladium inflatum ARSEF 3280	Hypocreales : Ophiocordycipitaceae	25.33	27.8	42 : 15 : 2 : 25	**KY924879.1**
Tolypocladium ophioglossoides	Hypocreales : Ophiocordycipitaceae	35.16	27.5	44 : 19 : 2 : 25	**KX455872.1**
Trichoderma asperellum B05	Hypocreales : Hypocreaceae	30.00	27.8	43 : 17 : 1 : 25 (1)[c]	**NC_037075.1**
Trichoderma atroviride ATCC 26799	Hypocreales : Hypocreaceae	32.76	28.2	51 : 21 : 2 : 27 (1)[c]	**MN125601.1**
Trichoderma gamsii KUC1747	Hypocreales : Hypocreaceae	29.30	28.3	46 : 18 : 2 : 26 (1)[c]	**KU687109.1**
Trichoderma hamatum	Hypocreales : Hypocreaceae	32.76	27.7	48 : 20 : 2 : 26 (1)[c]	**MF287973.1**
Trichoderma reesei QM9414	Hypocreales : Hypocreaceae	42.13	27.2	43 : 19 : 2 : 23 (2)[c]	**AF447590.1**
Neurospora crassa OR74A[d]	Sordariales : Sordariaceae	64.84	36.1	58 : 28 : - : 28	**KC683708.1**
Trichoderma harzianum CBS 226.95	Hypocreales : Hypocreaceae	27.63	27.6	44 : 18 : 2 : 24 (1)[c]	**MN564945** (this study)

[a]Based on the GenBank database, NCBI (datasets as of, January 2021).

[b]Non-existence of rRNAs were indicated with a dash (-) in the column.

[c]For the *Trichoderma* mitogenomes, a number of putative *trn* genes predicted within the protein-coding genes were indicated inside the parentheses.

[d]Used as an outgroup for the phylogenetic analysis.

Table S6. Gene components for the core-PCGs and rRNAs in the *Trichoderma* complete mitogenomes.

Gene (product) \ Species name	*T. harzianum* CBS 226.95	*T. reesei* QM9414[a]	*T. atroviride* ATCC 26799[a]	*T. gamsii* KUC1747[a]	*T. asperellum* B05[a]	*T. hamatum*[a]
	Gene placement (Start position – Stop position, Length (nt))[b]					
atp6 (ATP synthase F0 subunit 6)	10,695 - 11,477 (783)	3,601 - 4,380 (780)	14,161 - 14,943 (783)	7,876 - 8,658 (783)	24,539 - 25,321 (783)	2,377 - 3,159 (783)
atp8 (ATP synthase F0 subunit 8)	10,430 - 10,576 (147)	3,287 - 3,439 (153)	13,763 - 13,909 (147)	7,478 - 7,624 (147)	24,213 - 24,359 (147)	2,043 - 2,189 (147)
atp9 (ATP synthase F0 subunit 9)	25,409 - 25,603 (195)	18,927 - 19,130 (204)	31,610 - 31,834 (225)	-	10,876 - 11,100 (225)	19,266 - 19,490 (225)
cob (apocytochrome b)	3,254 - 4,423 (1,170)	27,380 - 30,923 (3,544)	3,498 - 6,182 (2,685)	1 - 1,170 (1,170)	17,024 - 18,193 (1,170)	26,342 - 27,511 (1,170)
cox1 (cytochrome c oxidase subunit 1)	5,102 - 6,688 (1,587)	31,523 - 40,232 (8,710)	6,801 - 9,672 (2,872)	1,790 - 3,382 (1,593)	18,848 - 20,434 (1,587)	28,132 - 30,995 (2,864)
cox2 (cytochrome c oxidase subunit 2)	26,883 - 27,632 (750)	21,830 - 23,704 (1,875)	32,009 - 32,758 (750)	25,061 - 25,810 (750)	11,269 - 12,018 (750)	19,665 - 22,856 (3,192)
cox3 (cytochrome c oxidase subunit 3)	14,058 - 14,867 (810)	6,815 - 7,624 (810)	17,536 - 18,345 (810)	11,878 - 12,078 (201)	27,675 - 29,602 (1,928)	5,837 - 7,764 (1,928)
nad1 (NADH dehydrogenase subunit 1)	7,194 - 8,303 (1,110)	40,831 - 41,973 (1,143)	10,326 - 11,435 (1,110)	3,993 - 5,150 (1,158)	21,062 - 22,171 (1,110)	31,638 - 32,747 (1,110)
nad2 (NADH dehydrogenase subunit 2)	23,140 - 24,807 (1,668)	16,531 - 18,315 (1,785)	29,342 - 31,009 (1,668)	22,394 - 24,061 (1,668)	8,600 - 10,267 (1,668)	16,990 - 18,657 (1,668)
nad3 (NADH dehydrogenase subunit 3)	24,808 - 25,221 (414)	18,316 - 18,729 (414)	31,010 - 31,423 (414)	24,062 - 24,475 (414)	10,268 - 10,681 (414)	18,658 - 19,071 (414)

nad4<br>(NADH dehydrogenase subunit 4)	8,565 - 9,941<br>(1,377)	1 - 1,470<br>(1,470)	11,647 - 13,104<br>(1,458)	5,362 - 6,819<br>(1,458)	22,313 - 23,770<br>(1,458)	171 - 1,628<br>(1,458)
nad4L<br>(NADH dehydrogenase subunit 4L)	628 - 897<br>(270)	24,021 - 24,320<br>(300)	292 – 561<br>(270)	26,099 - 26,368<br>(270)	12,329 - 13,618<br>(1,290)	23,141 - 23,410<br>(270)
nad5<br>(NADH dehydrogenase subunit 5)	897 - 2,888<br>(1,992)	24,320 - 26,398<br>(2,079)	561 - 2,627<br>(2,067)	26,368 - 28,434<br>(2,067)	14,075 - 16,135<br>(2,061)	23,410 - 25,470<br>(2,061)
nad6<br>(NADH dehydrogenase subunit 6)	15,111 - 15,863<br>(753)	7,964 - 8,734<br>(771)	18,638 - 19,390<br>(753)	12,378 - 13,130<br>(753)	192 – 698<br>(507)	8,058 - 8,810<br>(753)
rrnL<br>(large subunit ribosomal RNA)	16,743 - 21,455<br>(4,713)	9,721 - 14,234<br>(2,979)	20,245 - 24,937<br>(4,693)	14,188 - 16,603<br>(2,416)	-	9,924 - 14,632<br>(4,709)
rns<br>(small subunit ribosomal RNA)	12,055 - 13,557<br>(1,503)	4,858 - 6,247<br>(1,390)	15,514 - 17,017<br>(1,504)	9,249 - 10,750<br>(1,502)	25,676 - 27,177<br>(1,502)	3,753 - 5,241<br>(1,489)

[a]Based on the GenBank database, NCBI (datasets as of, January 2021).

[b]Non-existence of target genes in the mitogenome was indicated with a dash (-) in the column.

Table S7. Putative tRNA gene (*trn*) within the protein-coding gene of *Trichoderma* mitogenomes.

Species (GenBank assession no.)	Total number of putative *trn* gene	Replacement (predicted putative tRNA : Length (nt) : overlap position)
T. harzianum CBS 226.95 (GenBank accession no. **MN564945**, this study)	1	$tRNA^{Val}$: 72 bp (15,781 - 15,852)[b] : overlapped with the 3' end of the *nad6* gene (15,111 - 15,863)[c]
T. reesei QM9414 (GenBank accession no. **AF447590**)[a]	2	$tRNA^{Val}$: 72 bp (8,652 - 8,723)[b] : overlapped with the 3' end of the *nad6* gene (7,964 - 8,734)[c]<br>$tRNA^{Met}$: 72 bp (16,538 - 16,609)[b] : overlapped with the 5' end of the *nad2* gene (16,531 - 18,315)[c]
T. atroviride ATCC 26799 (GenBank accession no. **MN125601**)[a]	1	$tRNA^{Val}$: 72 bp (19,308 - 19,379)[b] : overlapped with the 3' end of the *nad6* gene (18,638 - 19,390)[c]
T. gamsii KUC1747 (GenBank accession no. **KU687109**)[a]	1	$tRNA^{Val}$: 72 bp (13,048 - 13,119)[b] : overlapped with the 3' end of the *nad6* gene (12,378 - 13,130)[c]
T. asperellum B05 (GenBank accession no. **NC_037075**)[a]	1	$tRNA^{Val}$: 72 bp (616 - 687)[b] : overlapped with the 3' end the *nad6* gene (192 – 698)[c]
T. hamatum (GenBank accession no. **MF287973**)[a]	1	$tRNA^{Val}$: 72 bp (8,728 - 8,799)[b] : overlapped with the 3' end of the *nad6* gene (8,058 - 8,810)[c]

[a]Based on the GenBank database, NCBI (datasets as of, January 2021).
[b]Start position - Stop position of a putative tRNA gene in the mitogenome.
[c]Start position - Stop position of each protein-coding gene in the mitogenome.

Table S8. Positions of detected group I intron loci on the mitogenomes of *Trichoderma* species.

Species (GenBank assession no.)	Sub type	Start - Stop position (Length (nt))[b]	Intronic Open reading frame (ORF)[c]	Note
T. harzianum CBS 226.95 (GenBank accession no. **MN564945**, this study)	IA	19,206 - 20,841 (1,636 bp)	*rps3*, *orf408*	*rrnL-i* (i.e., intron placed within the *rrnL*)
	IA	25,615 - 25,790 (176 bp)	*orf170*, GIY-YIG	-
T. reesei QM9414 (GenBank accession no. **AF447590**)[a]	IA	12,058 - 13,712 (1,655 bp)	*rps5*	*rrnL-i*
	IA	19,142 - 19,317 (176 bp)	*orf*	-
	IB	29,238 - 30,185 (948 bp)	-	*cob-i*2 (i.e., 2nd intron in the exon-intron structure of *cob*)
	IB	32,036 - 33,177 (1,142 bp)	-	*cox1-i*1 (i.e., 1st intron in the exon-intron structure of *cox1*)
	IB	33,521 - 34,590 (1,070 bp)	*orf*	*cox1-i*2 (i.e., 2nd intron in the exon-intron structure of *cox1*)
	IB	34,992 - 36,015 (1,024 bp)	-	*cox1-i*3 (i.e., 3rd intron in the exon-intron structure of *cox1*)
	IB	36,298 - 36,477 (180 bp)	-	*cox1-i*4 (i.e., 4th intron in the exon-intron structure of *cox1*)
	IB	37,672 - 38,712 (1,041 bp)	-	*cox1-i*5 (i.e., 5th intron in the exon-intron structure of *cox1*)
	IC2	20,553 - 20,784 (232 bp)	*orf*	-
	ID	23,141 - 23,290 (150 bp)	-	*cox2-i* (i.e., intron placed within the *cox2*)
	ID	28,844 - 28,966 (123 bp)	-	*cob-i*1 (i.e., 1st intron in the exon-intron structure of *cob*)
T. atroviride ATCC 26799 (GenBank accession no. **MN125601**)[a]	IA	22,709 - 24,328 (1,620 bp)	*rps3*, *orf455*	*rrnL-i*
	IB	4,042 - 4,989 (948 bp)	*orf320*, LAGLIDADG	*cob-i*1
	IB	5,404 - 5,638 (235 bp)	-	*cob-i*2
	IB	7,889 - 8,068 (180 bp)	*orf421*, GIY-YIG	*cox1-i* (i.e., intron placed within the *cox1*)
T. gamsii KUC1747 (GenBank accession no. **KU687109**)[a]	IA	16,622 - 18,230 (1,609 bp)	two *orf* genes	both for *rrnL-i*

T. asperellum B05	IA	4,056 - 5,686 (1,631 bp)	*orf459*	putative *rrnL-i*
(GenBank accession no.	IB	13,682 - 13,968 (287 bp)	-	-
**NC_037075**)[a]	IB	28,833 - 28,979 (147 bp)	*orf305*	*cox3-i* (i.e., intron placed within the *cox3*)
	IA	12,404 - 14,023 (1,620 bp)	*rps3*	*rrnL-i*
T. hamatum	IB	6,995 - 7,141 (147 bp)	LAGLIDADG	*cox3-i*
(GenBank accession no.	IB	22,120 - 22,306 (187 bp)	GIY-YIG	*cox2-i*2 (i.e., 2nd intron in the exon-intron structure of *cox2*)
**MF287973**)[a]	IB	29,220 - 29,399 (180 bp)	GIY-YIG	*cox1-i*
	IC2	19,786 - 20,017 (232 bp)	LAGLIDADG	*cox2-i*1 (i.e., 1st intron in the exon-intron structure of *cox2*)

[a]Based on the GenBank database, NCBI (datasets as of, January 2021).

[b]Detected on the RNAweasel webserver (http://megasun.bch.umontreal.ca/cgi-bin/RNAweasel/RNAweaselInterface.pl).

[c]Intronic ORF (i.e. intron-encoded ORF); *rps3*/*rps5*, genes coding for ribosomal proteins; *orf*, gene coding for hypotethical protein; LAGLIDADG or GIY-YIG, conserved structural motifs of homing endonuclease (HE); Non-existence of genes were indicated with a dash (-).

Table S9. Comparison of the mitochondrial genomic features between *T. harzianum* CBS 226.95 and *T. harzianum* HB324.

Genomic features	***T. harzianum*** **CBS 226.95**	***T. harzianum*** **HB324**[a]	Note
GenBank accession no.	**MN564945** (this study)	**MT263519**[b]	
Total size (bp)	27,632	32,277	
GC- content (%)	27.55	27.74	
AT- / GC- skew	(-)0.004 / 0.098	0.008 / 0.112[c]	calculated in this study
tRNAs / rRNAs	24 (1)[d] / 2	28 (-)[d] / 2	putative *trn* genes that calculated in this study[d]
Genome identity (%)[e]	used as query	92.23 (94.0%)[c]	calculated in this study

Comparison of gene components (core PCGs)

Gene	***T. harzianum*** **CBS 226.95** (Length (bp))[f]	***T. harzianum*** **HB324**[a] (Length (bp))[f]	Re-calculated- and/or modified annotations of the ***T. harzianum*** **HB324** mitogenome in this study
atp6	783	783	-
atp8	147	147	modified as: 26,169 - 26,315 (147)
atp9	195	219	re-calculation of the size (bp): 10,957 - 11,181 (225)
cob	1,170	*cob_a* (396) - *cob-i* (123) - *cob_b* (759)	modified as: *cob*_exon1 (16,541 - 16,936 (396)) - *cob-i* (16,937 - 18,139 (1,203)) - *cob*_exon2 (18,140 - 18,595 (456))

cox1	1,587	*cox1_a* (213) - *cox1-i* (1,350) - *cox1_b* (1,371)	modified as: *cox1*_exon1 (19,593 - 19,805 (213)) - *cox1-i* (19,806 - 21,056 (1,251)) - *cox1*_exon2 (21,057 - 22,430 (1,374))
cox2	750	*cox2_a* (84) - *cox2-i* (232) - *cox2_b* (657)	modified as: 12,734 - 13,399 (666)
cox3	810	495	-
nad1	1,110	1,089	-
nad2	1,668	1,668	-
nad3	414	405	re-calculation of the size (bp): 10,353 - 10,766 (414)
nad4	1,377	*nad4_a* (1,419)<br>*nad4_b* (216)<br>(non-existence of the intron *nad4-i* between *nad4_a* and *nad4_b*)	modified as: 24,223 - 25,680 (1,458)
nad4L	270	267	re-calculation of the size (bp): 13,888 - 14,157 (270)
nad5	1,992	1,329	-
nad6	753<br>(putative *trnV*^{Val})	645	modified as: 30,863 - 31,252 (390)

[a]Based on the publication of the *T. harzianum* HB324 mitogenome [68].

[b]Unverified nucleotide sequences that deposited in GenBank database, NCBI (dataset as of, January 2021).

[c]Calculated in this study, using the 'unverified nucleotide sequences' of the *T.harzianum* HB324 mitogenome[b].
[d]A number of putative *trn* genes that predicted within the protein-coding genes (details of *trn* positions in **Table S7**).
[e]Using BLAST searches between *T. harzianum* CBS 226.95 and *T.harzianum* HB324; values of query coverage (%) for the sequence identity were indicated inside the parentheses.
[f]Gene size (Length (bp)) or CDS(exon)/intron size (Length (bp)) under the exon-intron structure

Table S10. Estimated p-distance values among the Hypocreales mitogenomes.[a]

Strain[b]	S01	S02	S03	S04	S05	S06	S07	S08	S09	S10	S11	S12	S13	S14	S15	S16	S17	S18	S19[c]
**S01**	0.000																		
**S02**	0.055	0.000																	
**S03**	0.055	0.005	0.000																
**S04**	0.057	0.023	0.023	0.000															
**S05**	0.057	0.024	0.024	0.015	0.000														
**S06**	0.057	0.065	0.066	0.068	0.069	0.000													
**S07**	0.099	0.121	0.122	0.124	0.124	0.127	0.000												
**S08**	0.104	0.104	0.105	0.105	0.106	0.118	0.143	0.000											
**S09**	0.154	0.158	0.158	0.157	0.157	0.166	0.162	0.167	0.000										
**S10**	0.155	0.158	0.157	0.158	0.158	0.165	0.153	0.157	0.130	0.000									
**S11**	0.156	0.161	0.160	0.161	0.161	0.166	0.178	0.168	0.176	0.183	0.000								
**S12**	0.155	0.158	0.159	0.157	0.158	0.164	0.176	0.167	0.172	0.179	0.053	0.000							
**S13**	0.135	0.135	0.135	0.134	0.134	0.148	0.163	0.157	0.164	0.165	0.154	0.152	0.000						
**S14**	0.145	0.145	0.144	0.144	0.142	0.157	0.170	0.164	0.172	0.176	0.160	0.156	0.072	0.000					
**S15**	0.159	0.164	0.164	0.165	0.166	0.172	0.178	0.173	0.176	0.188	0.169	0.169	0.173	0.180	0.000				
**S16**	0.161	0.162	0.163	0.165	0.164	0.168	0.180	0.174	0.177	0.182	0.172	0.170	0.172	0.181	0.143	0.000			
**S17**	0.144	0.146	0.146	0.143	0.146	0.153	0.162	0.158	0.168	0.169	0.167	0.165	0.155	0.166	0.156	0.163	0.000		
**S18**	0.139	0.141	0.142	0.143	0.143	0.150	0.161	0.152	0.163	0.169	0.162	0.164	0.152	0.156	0.161	0.162	0.111	0.000	
**S19**[c]	0.232	0.230	0.230	0.231	0.232	0.238	0.241	0.231	0.241	0.247	0.240	0.237	0.240	0.246	0.239	0.240	0.225	0.228	0.000

[a]Based on the pairwise alignments of mitogenomes among Hypocreales species.

[b]As the descriptions in **Figure 4B**; **S01**, *Trichoderma harzianum* CBS 226.95 (GenBank accession no. **MN564945**, in this study); **S02**, *T. atroviride* ATCC

26799 (GenBank accession no. **MN125601**); **S03**, *T. gamsii* KUC1747 (GenBank accession no. **KU687109**); **S04**, *T. asperellum* B05 (GenBank accession no. **NC_037075**); **S05**, *T. hamatum* (GenBank accession no. **MF287973**); **S06**, *T. reesei* QM9414 (GenBank accession no. **AF447590**); **S07**, *T. harzianum* HB324 (GenBank accession no. **MT263519**); **S08**, *Hypomyces aurantius* (GenBank accession no. **KU666552**); **S09**, *Hirsutella thompsonii* ARSEF 9457 (GenBank accession no. **MH367294**); **S10**, *Ophiocordyceps sinensis* (GenBank accession no. **KY622006**); **S11**, *Cordyceps militaris* EFCC-C2 (GenBank accession no. **KF432176**); **S12**, *Beauveria bassiana* (GenBank accession no. **EU371503**); **S13**, *Epichloe typhina* E8 (GenBank accession no. **KX066185**); **S14**, *Metarhizium anisopliae* ME1 (GenBank accession no. **AY884128**); **S15**, *Acremonium chrysogenum* ATCC 11550 (GenBank accession no. **KF757229**); **S16**, *Clonostachys rosea* 6792 (GenBank accession no. **KU668563**); **S17**, *Nectria cinnabarina* 5175 (GenBank accession no. **KT731105**); **S18**, *Fusarium oxysporum* F11 (GenBank accession no. **AY945289**); **S19**, *Neurospora crassa* OR74A (GenBank accession no. **KC683708**).
[c]Used as an outgroup for the phylogenetic analysis in **Figure 4A**.

Table S11. Ka/Ks ratios for the 13 core protein-coding genes in the Hypocreaceae mitogenomes.

Gene pairs between[a]		Ka (nonsynonymous substitution rate) / Ks (synonymous substitution rate) ratio[b]												
		atp6	*atp8*	*cob*	*cox1*	*cox2*	*cox3*	*nad1*	*nad2*	*nad3*	*nad4*	*nad4L*	*nad5*	*nad6*
**S01**	**S02**	0.032	0.000	0.069	0.047	0.048	0.861	0.062	0.120	0.127	0.158	0.000	0.020	0.024
**S01**	**S08**	0.123	0.109	0.108	0.066	0.064	0.129	0.211	0.108	0.266	0.118	0.010	0.039	0.048
**S01**	**S04**	0.034	0.000	0.045	0.043	0.059	0.861	0.050	0.121	0.125	0.188	0.325	0.035	0.036
**S01**	**S03**	0.032	0.000	0.065	0.049	0.053	0.455	0.063	0.148	0.116	0.162	0.000	0.020	0.023
**S01**	**S05**	0.034	0.000	0.047	0.058	0.089	0.861	0.050	0.116	0.186	0.179	0.000	0.036	0.065
**S01**	**S06**	0.052	0.144	0.070	0.105	0.011	1.127	0.054	0.062	0.111	0.117	0.000	0.018	0.041
**S01**	**S07**	0.242	0.221	0.174	0.069	0.055	0.024	0.057	0.130	0.066	0.149	0.015	0.107	1.249
**S02**	**S08**	0.128	0.139	0.113	0.080	0.080	0.901	0.191	0.078	0.265	0.150	0.014	0.035	0.055
**S02**	**S04**	0.026	0.000	0.046	0.023	0.066	0.000	0.023	0.143	0.146	0.151	0.774	0.020	0.043
**S02**	**S03**	0.000	0.000	0.141	0.221	0.000	0.282	0.000	0.231	0.000	0.000	0.000	0.000	0.000
**S02**	**S05**	0.016	0.000	0.048	0.062	0.066	0.000	0.022	0.142	0.284	0.198	0.000	0.020	0.096
**S02**	**S06**	0.027	0.144	0.114	0.105	0.049	0.113	0.034	0.099	0.124	0.142	0.000	0.000	0.059
**S02**	**S07**	0.082	0.154	0.128	0.070	0.040	0.781	0.074	0.137	0.163	0.186	0.019	0.087	1.078
**S08**	**S04**	0.121	0.139	0.092	0.069	0.069	0.799	0.175	0.085	0.315	0.146	0.167	0.036	0.051
**S08**	**S03**	0.128	0.139	0.114	0.079	0.080	0.558	0.188	0.090	0.254	0.152	0.015	0.036	0.052
**S08**	**S05**	0.111	0.107	0.088	0.072	0.078	0.799	0.178	0.088	0.310	0.134	0.014	0.038	0.077
**S08**	**S06**	0.117	0.095	0.091	0.125	0.062	1.266	0.190	0.084	0.295	0.120	0.015	0.036	0.051
**S08**	**S07**	0.187	0.229	0.156	0.058	0.074	0.059	0.155	0.108	0.255	0.141	0.024	0.098	1.721
**S04**	**S03**	0.028	0.000	0.014	0.034	0.060	0.359	0.021	0.259	0.146	0.144	0.651	0.020	0.052
**S04**	**S05**	0.011	0.000	0.000	0.063	0.155	0.000	0.000	0.190	0.309	0.059	0.774	0.000	0.267

**S04**	**S06**	0.035	0.144	0.071	0.103	0.044	0.100	0.023	0.106	0.110	0.177	0.765	0.013	0.050
**S04**	**S07**	0.091	0.154	0.117	0.065	0.047	0.781	0.064	0.136	0.146	0.181	0.121	0.095	1.249
**S03**	**S05**	0.015	0.000	0.015	0.068	0.056	0.282	0.023	0.234	0.284	0.188	0.000	0.021	0.111
**S03**	**S06**	0.026	0.144	0.086	0.098	0.039	0.189	0.034	0.124	0.113	0.145	0.000	0.000	0.054
**S03**	**S07**	0.082	0.154	0.130	0.070	0.045	0.000	0.071	0.138	0.148	0.188	0.018	0.086	1.108
**S05**	**S06**	0.028	0.092	0.078	0.113	0.067	0.089	0.022	0.115	0.179	0.163	0.000	0.012	0.090
**S05**	**S07**	0.089	0.130	0.112	0.074	0.055	0.781	0.060	0.135	0.235	0.177	0.019	0.099	1.222
**S06**	**S07**	0.105	0.224	0.161	0.091	0.032	1.294	0.079	0.121	0.188	0.154	0.016	0.097	1.318

[a]As the descriptions in **Figure 5**; **S01**, *Trichoderma harzianum* CBS 226.95 (GenBank accession no. **MN564945**, in this study); **S02**, *T. atroviride* ATCC 26799 (GenBank accession no. **MN125601**); **S03**, *T. gamsii* KUC1747 (GenBank accession no. **KU687109**); **S04**, *T. asperellum* B05 (GenBank accession no. **NC_037075**); **S05**, *T. hamatum* (GenBank accession no. **MF287973**); **S06**, *T. reesei* QM9414 (GenBank accession no. **AF447590**); **S07**, *T. harzianum* HB324 (GenBank accession no. **MT263519**); **S08**, *Hypomyces aurantius* (GenBank accession no. **KU666552**).

[b]Based on the pairwise alignments of target genes (one-to-one gene pairs) among Hypocreaceae species.

Table S12. Prediction of codon sites evolving under positive diversifying selection on the MEME[a] approach.

Target Gene[b] (No. of codons in the alignments)	No. of sites	Position of selected codon sites[c] (α, β^+ (corresponding *p*-value))
atp6 (268)	7	**13** (0.000, 18.199 (0.0222)), **15** (0.000, 1.173 (0.0341)), **65** (0.750, 40.034 (0.0640)), **67** (0.000, 1.974 (0.0848)), **78*** (0.000, 2.208 (0.0333)), **138*** (0.000, 3.855 (0.0753)), **152** (0.033, 23.518 (0.0758))
atp8 (57)	0	None
cob (415)	13	**21** (0.000, 15.350 (0.0052)), **42*** (0.189, 8.701 (0.0302)), **60** (0.626, 20.479 (0.0292)), **119*** (0.078, 23.030 (0.0698)), **171*** (0.371, 12.485 (0.0944)), **172*** (0.379, 10000.00 (0.0262)), **198** (0.000, 4.064 (0.0844)), **239** (0.472, 49.307 (0.0777)), **254*** (0.423, 63.201 (0.0214)), **280*** (0.000, 44.312 (0.0431)), **301*** (0.282, 7.420 (0.0047)), **316*** (0.362, 119.841 (0.0128)), **319*** (0.000, 3.578 (0.0193))
cox1 (963)	20	**30** (1.026, 1876.100 (0.0739)), **167** (0.229, 3.887 (0.0769)), **190** (0.000, 26.795 (0.0380)), **221*** (0.685, 144.256 (0.0623)), **232*** (0.322, 36.886 (0.0273)), **233*** (0.736, 40.641 (0.0595)), **236*** (1.091, 51.491 (0.0715)), **238*** (0.536, 7.710 (0.0797)), **239*** (0.000, 63.672 (0.0002)), **257** (0.000, 0.669 (0.0954)), **401** (0.000, 17.295 (0.0140)), **405** (0.463, 22.903 (0.0623)), **407*** (0.431, 16.133 (0.0586)), **437*** (0.397, 109.698 (0.0981)), **438*** (0.000, 20.317 (0.0148)), **574*** (0.257, 65.940 (0.0320)), **584** (0.053, 12.035 (0.0287)), **606** (0.057, 10.518 (0.0380)), **617** (0.179, 1.937 (0.0662)), **644** (0.000, 19.901 (0.0054))
cox2 (570)	5	**4*** (0.000, 2.928 (0.0981)), **11*** (0.138, 9.066 (0.0530)), **52** (0.129, 11.107 (0.0730)), **76** (0.069, 29.087 (0.0903)), **513*** (0.000, 19.254 (0.0011))
cox3 (272)	5	**4** (0.000, 2.996 (0.0489)), **73*** (0.657, 11.552 (0.0223)), **160*** (0.179, 11.768 (0.0708)), **167** (0.000, 0.653 (0.0920)), **206*** (0.000, 1243.664 (0.0078))

nad1 (395)	3	**53** (0.000, 4.841 (0.0421)), **211** (0.000, 32.545 (0.0642)), **286** (0.066, 3.992 (0.0245))
nad2 (907)	11	**113*** (0.430, 82.307 (0.0028)), **134** (0.113, 24.804 (0.0679)), **142*** (0.774, 735.424 (0.0023)), **311*** (0.000, 35.829 (0.0894)), **405*** (0.217, 14.711 (0.0004)), **507** (0.147, 13.299 (0.0609)), **553** (0.261, 37.995 (0.0994)), **612*** (0.000, 3.057 (0.0597)), **625*** (0.732, 159.019 (0.0426)), **741** (0.000, 6.965 (0.0012)), **750** (0.279, 22.155 (0.0275))
nad3 (452)	2	**17** (0.037, 45.692 (0.0295)), **50** (0.000, 2.539 (0.0192))
nad4 (592)	6	**20*** (0.000, 154.099 (0.0324)), **272*** (0.000, 2.081 (0.0315)), **369** (0.000, 2.847 (0.0672)), **392*** (0.030, 7.754 (0.0169)), **521** (0.000, 37.361 (0.0186)), **544** (0.000, 8.317 (0.0402))
nad4L (472)	4	**89** (0.000, 3.010 (0.0992)), **91*** (0.000, 30.669 (0.0595)), **116*** (0.000, 21.641 (0.0182)), **122*** (0.086, 32.703 (0.0730))
nad5 (957)	11	**40** (0.347, 21.747 (0.0171)), **51** (0.169, 40.739 (0.0850)), **70** (0.178, 23.229 (0.0393)), **177** (0.000, 9.669 (0.0089)), **264** (0.000, 2.781 (0.0496)), **420*** (0.170, 12.355 (0.0549)), **556*** (0.642, 6.605 (0.0983)), **567*** (0.000, 11.648 (0.0501)), **573*** (0.230, 344.924 (0.0074)), **690*** (0.499, 6.432 (0.0818)), **727*** (0.000, 2.228 (0.0280))
nad6 (598)	4	**20*** (0.017, 6.186 (0.0898)), **52*** (0.069, 11.053 (0.0542)), **238** (0.232, 4.150 (0.0369)), **284*** (0.000, 7.349 (0.0712))

[a]Using the Mixed Effects Model of Evolution (MEME) [54].

[b]Based on the phylogeny in **Figure 4A**.

[c]Detected codon sites under the episodic diversifying positive selection at p-values < 0.1; α, site-specific synonymous substitution rate; β^{+}, site-specific non-synonymous substitution rate for the positive selection; sites that predicted within the *Trichoderma* clades were indicated by an asterisk.
