# Supplementary figures and images for: An Update on Trichoderma Mitogenomes: Complete De Novo Mitochondrial Genome of the Fungal Biocontrol Agent Trichoderma harzianum (Hypocreales, Sordariomycetes), an Ex-Neotype Strain CBS 226.95, and Tracing the Evolutionary Divergences of Mitogenomes in Trichoderma
Source: Microorganisms. 2021 Jul 23;9(8):1564. doi: 10.3390/microorganisms9081564 (PMC8401334; doi:10.3390/microorganisms9081564)

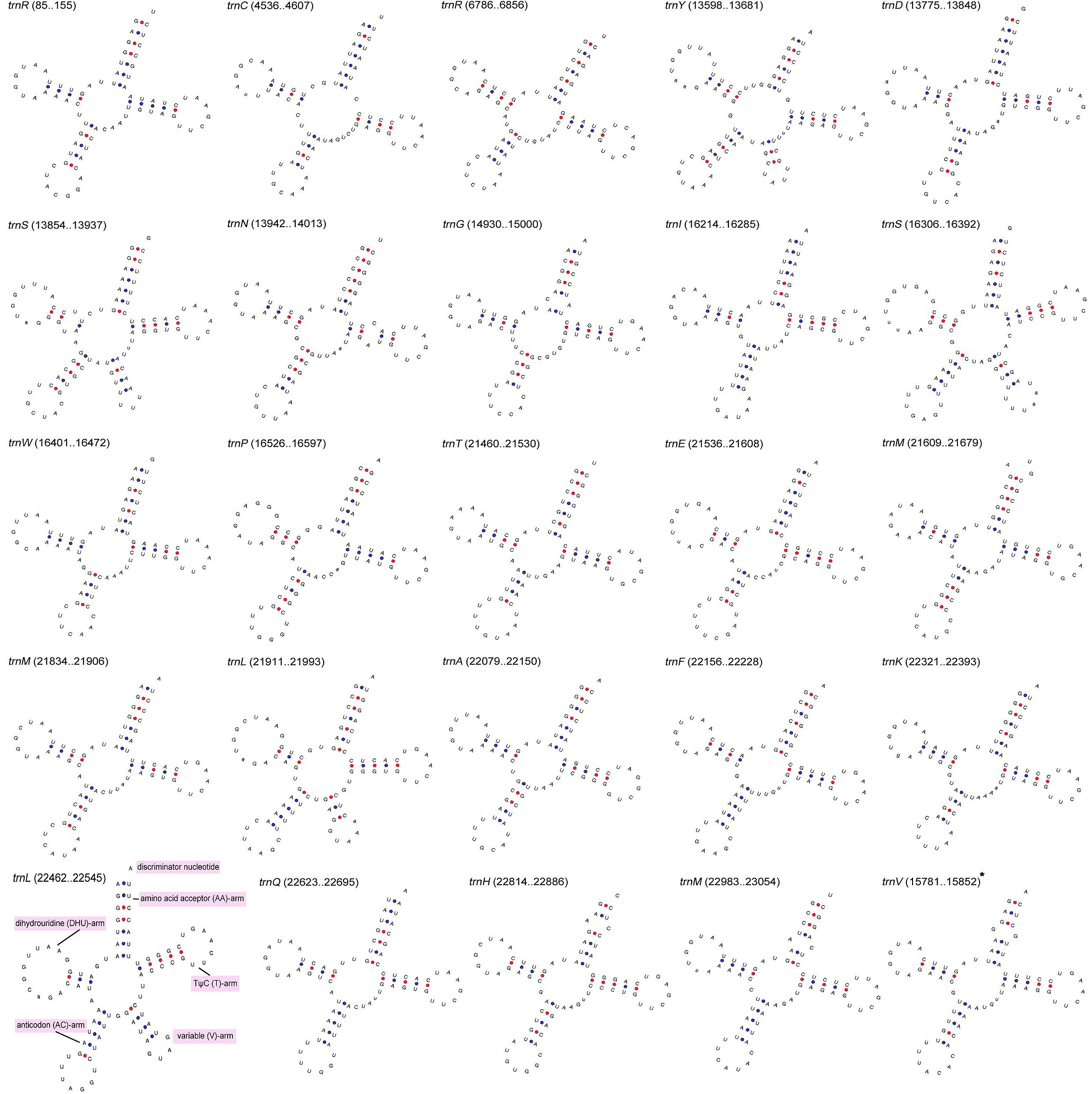

Supplement: Supplementary file 1 [file microorganisms-09-01564-s001.zip › microorganisms-1285849-supplementary/Figure_S1.jpg]

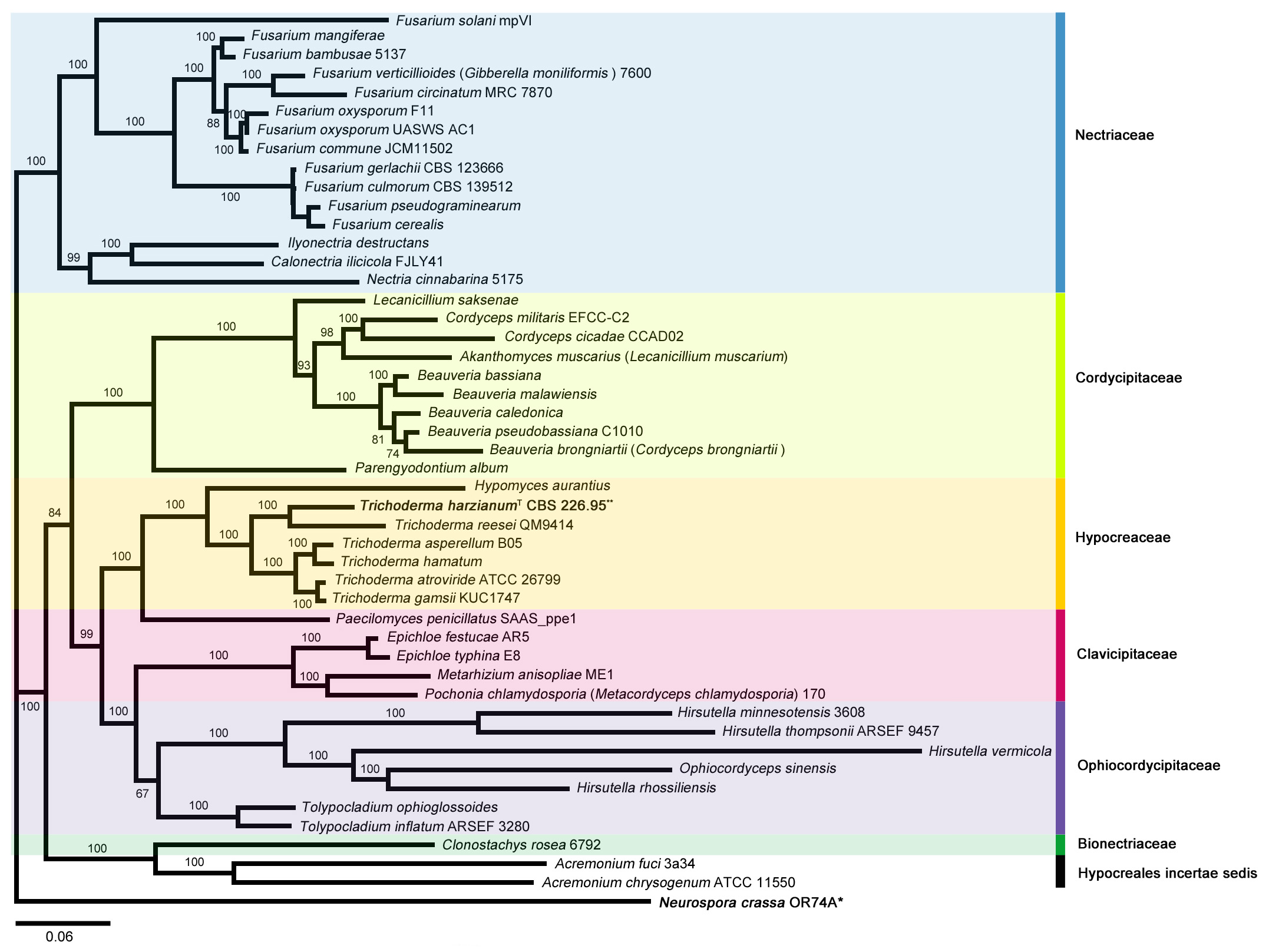

Supplement: Supplementary file 1 [file microorganisms-09-01564-s001.zip › microorganisms-1285849-supplementary/Figure_S2.jpg]
